# A review on the ethnopharmacology, metabolites, pharmacological uses, and toxicology of *Ficus hirta (Moraceae)* Vahl

**DOI:** 10.3389/fphar.2025.1545348

**Published:** 2025-04-24

**Authors:** Minghui Chen, Jiao Liu, Sihua Zou, Jingquan Yuan, Jiyu Pan, Zhenmei Lin, Yanchun Wu, Bei Zhou

**Affiliations:** ^1^ Guangxi University of Chinese Medicine, Nanning, China; ^2^ Guangxi Key Laboratory of Efficacy Study on Chinese Materia Medicine, Nanning, China

**Keywords:** *Ficus hirta (Moraceae)* Vahl, botanical characterization, traditional medicine, phytochemistry, pharmacology, toxicology

## Abstract

*Ficus hirta* Vahl, a member of the Moraceae family and genus *Ficus*, is a traditional medicinal plant with diverse metabolites. Traditionally, *Ficus hirta* Vahl has been used in Asian countries to treat various ailments including indigestion, loss of appetite, cold, pneumonia, tuberculosis, cough, asthma, excessive sweating, backache, bruises, arthritis, liver diseases, skin conditions, gynecological disorders, and pediatric illnesses. As a botanical drug with both medicinal and nutritional applications, *Ficus hirta* Vahl is an integral part of China’s multi-ethnic medical system, serving as a tonic for many years. Approximately 130 chemical metabolites have been identified from *Ficus hirta* Vahl to date, including flavonoids, phenylpropanoids, phenolics, phenol glycosides, terpenoids, sterols, quinones, and esters. The bioactive properties of *Ficus hirta* Vahl extracts include immune modulation, enhancement of digestive system function, antitussive and antiasthmatic effects, as well as antibacterial, anti-inflammatory, antioxidant, anti-aging, hepatoprotective, anti-radiation, and antitumor activities. Toxicological assessments confirmed the safety and nontoxicity of *Ficus hirta* Vahl A thorough review of the literature underscores the significant potential of *Ficus hirta* Vahl across multiple domains such as medicine, food, and agriculture. However, research on *Ficus hirta* Vahl remains limited, necessitating further investigation into its pharmacokinetics and mechanisms of action.

## 1 Introduction

*Ficus hirta (Moraceae)* Vahl, termed “*Wuzhi Maotao*” in Chinese, is a traditional botanical drug from the genus *Ficus* and occupies a prominent position in the Moraceae family (Lu et al., 2022; Zhong and Xu, 2000). *Ficus hirta* Vahl is extensively recorded in literature, demonstrating the significance of its use as a medicinal plant. Owing to its long history of use and extensive distribution, *Ficus hirta* Vahl has taken on numerous aliases. After literature analysis, it was determined that the name originated from the coarse leaves of *Ficus* of the Moraceae family (Singh and Sharma, 2023). Roots, used either fresh or dried, are the most commonly used plant parts used in herbal medicine. After a thorough examination of classical Chinese herbal medicine books and ethnopharmacology data, *Ficus hirta* Vahl was found to be used either alone or in combination with other botanical drugs to treat a variety of diseases, including loss of appetite, indigestion, hypogalactia, tuberculosis, cough, asthma, and edema (Lin et al., 2013; Bhambhani et al., 2021). Furthermore, the fruit of *Ficus hirta* Vahl is nutrient-rich, nourishes body fluids, acts as a laxative, stimulates lactation, and enhances immunity. It is also used to treat constipation and hypolactation (Wang and Chen, 2016). Phytochemical studies revealed that *Ficus hirta* Vahl is an essential source of flavonoids, phenylpropanoids, phenolics, phenolic glycosides, and other metabolites. The main metabolites of *Ficus hirta* Vahl are listed in Table 1. It has various pharmacological effects, including antibacterial (Chen et al., 2020b), anti-inflammatory (Ye et al., 2020), antioxidant (Ye et al., 2016; Yi et al., 2013), hepatoprotective (Du, 2021), and immunoregulatory (Yang et al., 2015) activities.

**TABLE 1:** The main metabolites in *Ficus hirta* Vahl are listed in this table https://data.rbge.org.uk/herb/E00364493.

Category		NO.	Phytochemicals	Reference
Flavonoids		1	(+/−)-Naringenin	Za et al. (2008)
		2	blumeatin	Ye et al. (2021)
		3	(8*R*)-4,5′-dihydroxy-8-hydroxymehtyl-3′-methoxydeoxybenzoin	Cheng et al. (2017)
		4	apigenin	Yang et al. (2013)
		5	luteolin	Qin et al. (2020)
		6	norartocarpetin	Za et al. (2008)
		7	kaempferol	Za et al. (2008)
		8	quercetin	Za et al. (2008)
		9	yinyanghuo D	Ye et al. (2021)
		10	kuwanon T	Ye et al. (2021)
		11	cyclomorusin	Ye et al. (2021)
		12	acacetin	Qin et al. (2020)
		13	diosmetin	Za et al. (2008)
		14	3-acetyl-3, 5, 4′-trihydroxy-7-methoxyflavone	Ya et al. (2010)
		15	tricin	Zheng et al. (2013a)
		16	5,3′,4′-trihydroxy-3,7-dimethoxyflavone	Za et al. (2008)
		17	3, 5, 4′-trihydroxy-6, 7, 3′-trimethoxyflavone	Zheng et al. (2013b)
		18	5-hydroxy-3,7,4′-trimethoxyflavone	Za et al. (2008)
		19	5-hydroxy-4′,6,7,8-tetramethoxy flavone	Li et al. (2006)
		20	4′,5,6,7,8-pentamethoxy flavone	Zheng et al. (2013b)
		21	3,7-Dihydroxy-3′,4′,5′-trimethoxyflavone	Ye (2021)
		22	5-hydroxy-3,6,7-trimethoxy-2-(4- methoxyphenyl) chromen-4-one	Ye et al. (2019)
		23	vitexicarpin	Ye (2021)
		24	cyclomorusin	Zheng et al. (2013a)
		25	pinocembroside	Za et al. (2008)
		26	naringenin-7-*O*-*β*-D-glucoside	Wan et al. (2017)
		27	astragalin	Za et al. (2008)
		28	rutin	Technology and Fruits (2020)
		29	quercetin 3-*O*- [ (6-*O*-E-sinapoyl) -*β*-D-glucopyranosyl]- (1 →2) -*β*-D-glucopyranoside	Zheng et al. (2013b)
		30	pinocembrin-7-*O*-*β*-D-glucoside	Wan et al. (2017)
		31	acacetin 7-*O*-*β*-D-glucopyranoside	Za et al. (2008)
		32	luteolin-7-*O*-*β*-D-glucopyranoside	Za et al. (2008)
		33	vitexin	Zhao et al. (2008)
		34	nepetrin	Ye et al. (2020)
		35	hesperidin	Ya et al. (2010)
Phenylpropanoids	Simple phenylpropanoids	36	trans-4-Hydroxycinnamic acid	Chen (2017)
		37	2, 3-dihydroxy-l- (4-hydroxy-3-methoxyphenyl) -propan-1-one	Chen (2017)
		38	*β*-hydroxy propiovanillone	Chen (2017)
		39	7-*O*-ethylguaiacylglycerol	Chen (2017)
		40	(2′*S*) -3- [2, 3-dihydro-6-hydroxy-2-(l-hydroxy-l-methylethyl) -5-benzofuranyl] methyl propionate 40	Cheng et al. (2017)
		41	Chlorogenic acid methyl ester	Zheng et al. (2013a)
		42	1-*O*-trans-cinnamoyl-*β*-D-glucopyranosyl-(1→6)-*β*-D-glucopyranoside	Wan et al. (2016)
		43	ferulaldehyde	Cheng et al. (2017)
		44	2- [4- (3-hydroxypropyl) -2-methoxyphenoxy]-propane-1, 3-diol	Cheng et al. (2017)
		45	ficuscarpanoside A	Cheng et al. (2017)
		46	(+) - (7*R*, 8*R*)-4-hydroxy-3, 3′, 5′-trimethoxy-8′, 9′-dinor-8, 4′-oxyneoligna-7, 9-diol-7′-aldehyde	Cheng et al. (2017)
		47	(−) - (7*R*, 8*R*)-4-hydroxy-3, 3′, 5′-trimethoxy-8′, 9′-dinor-8, 4′-oxyneoligna-7, 9-diol-7′-aldehyde	Cheng et al. (2017)
		48	methyl chlorogenate	Zheng et al. (2013b)
	Coumarins	49	umbelliferone	Za et al. (2008)
		50	esculetine	Yang et al. (2013)
		51	ispsoralen	Lin et al. (2000)
		52	psoralen	Ye, et al. (2021)
		53	bergapten	Lin et al. (2000)
		54	7- (2′, 3′-dihydroxy-3′-methylbutoxy) -coumarin	Chen (2017)
		55	nodakenetin	Chen (2017)
		56	(*E*) -suberenol	Ye et al. (2021)
		57	meranzin hydrate	Zheng et al. (2013a)
		58	carnation	Deng et al. (2018)
		59	5-methoxyl-4, 2′-epoxy-3-(4, 5′-dihydroxypheny) -linearpyrano coumarin	Ya et al. (2010)
		60	l′-*O*-*β*-D- glucopyranosyl (2*R*, 3*S*) -3-hydroxynodakenetin	Chen (2017)
		61	(1′*S*) -6- (2′-hydroxy-l′-*O*-*β*-D-glucopyranosyl) -7-hydroxy coumarin	Chen (2017)
	Simple lignans	62	evofolin-B	Cheng et al. (2017)
	Bisepoxylignans	63	syringaresinol	Technology and Fruits (2020)
		64	(−)-pinoresinol	Wan et al. (2016)
		65	pinoresinol	Cheng et al. (2017)
		66	syringaresinol	Cheng et al. (2017)
	Benzofuran lignans	67	(*Z*) -3- [5- (6-methoxy) benzofuranyl] propenoic acid	Ye et al. (2021)
		68	(*Z*) -isopsoralic acid-1→6-*O*-*β*-D-glucopyranoside	Ye et al. (2021)
		69	(*Z*)-3- [5- (6-*O*-*β*-D-glucopyranosyl) benzofuranyl] methyl propenoate	Ye et al. (2021)
		70	(*E*) -3- [5- (6-methoxy) benzofuranyl] propenoic acid	Ye et al. (2021)
		71	(*E*) -isopsoralicacid-1→6-*O*-*β*-D-glucopyranoside	Ye et al. (2021)
		72	(*E*) -3- [5- (6-hydroxy) benzofuranyl] propenoic acid	Ye et al. (2021)
		73	methylcnidioside A	Ye et al. (2021)
		74	3- [6- (5-*O*-*β*-D-glucopyranosyl) 4-benzofuranyl] methyl propionate	Ye et al. (2021)
		75	3- [6- (5-*O*-*β*-D-glucopyranosyl) 4-benzofuranyl] methyl propionate	Cheng et al. (2017)
		76	(7*R*, 8*S*) -ficusal	Cheng et al. (2017)
Phenolics		77	cresols	Yang et al. (2023)
		78	hydroquinone	Yang et al. (2023)
		79	4-Hydroxybenzoic acid	Zheng et al. (2013a)
		80	methyleugenol	Zheng et al. (2013b)
		81	vanillin	Chen (2017)
		82	vanillic acid	Chen (2017)
		83	p-hydroxybenzoic acid	Chen (2017)
		84	syringic acid	Chen (2017)
		85	ficuglucoside	Chen (2017)
		86	evofolin B	Chen (2017)
		87	(8*R*) −4, 5′dihydroxy-8 hydroxymehtyl-3′-methoxy deoxybenzoin	Chen (2017)
		88	methyl chlorogenic acid	Chen (2017)
Phenol glycosides		89	3,4-dimethoxyphenyl-1-*O*-*β*-D-glucopyranoside	Cheng et al. (2017)
		90	3,4,5-trimethoxy phenoltetraacetyl-*β*-D-glucopyranoside	Cheng et al. (2017)
		91	ficuglucoside	Cheng et al. (2017)
		92	3,4,5-trimethoxybenzyl *β*-D-glucopyranoside	Cheng et al. (2017)
		93	(2*S*)-1-O-*β*-D-glucopyranosyl-2-*O*-(2-methoxy-4-phenylaldehyde)propane-3-ol	Cheng et al. (2017)
		94	(2*R*)methyl 2-*O*-*β*-Dglucopyranosyl-2-phenyl acetate	Wan et al. (2016)
		95	2-phcnylethyl-*O*-*β*-D-glucoside	Wan et al. (2016)
		96	benzyl-*β*-D-glucopyranoside	Wan et al. (2016)
		97	2-phenvlethyl-*O*-*β*-vicianoside	Wan et al. (2016)
		98	phenyl-*β*- D-glucopyranoside	Zhao et al. (2008)
Terpenoids		99	(+)-Cedrol	Liu et al. (2004a)
		100	lupeol	Chen (2017)
		101	*β*-Amyrin	Chen (2017)
		102	oleanic acid	Chen (2017)
		103	taraxerol	Thien et al. (2019)
		104	olean-12-ene-11*α*-methoxy -3*β*-acetate	Chen (2017)
		105	3*β*-acetoxy-11*α*-methoxy-12-ursene	Thien et al. (2019)
		106	alpha-Amyrin	Wan et al. (2017)
		107	*α*-amyrin	Chen (2017)
		108	ursolicacid	Chen (2017)
		109	3*β*-acetoxy-11*α*-hydroxy-12-ursene	Thien et al. (2019)
		110	3*β*-hydroxy-11-oxo-olean-12-enyl-3-stearate	Thien et al. (2019)
Sterols		111	(24*S*)-24-ethylcholesta-3*β*,5*α*,6*β*-triol	Chen (2017)
		112	*β*-sitosterol	Chen (2017)
		113	24-methylenecycloartanol	Chen (2017)
		114	stigmasta-5,22-diene-3*β*,7*α*-diol	Chen (2017)
		115	7*β*-hydroxy-4,22-stigmastadien-3-one	Chen (2017)
		116	7-ketositosterol	Chen (2017)
		117	7*α*-hydroxysitosterol	Chen (2017)
		118	7-ketositosterol-3-*O*-*β*-D-glucopyranoside	Chen (2017)
		119	stigmasterol	Li et al. (2006)
		120	*β*-daucosterol	Chen (2017)
		121	*β*-sitosteryl-3*β*-D-glucopyranoside-6′-palmitate	Chen (2017)
		122	daucostero	Li et al. (2006)
Quinones		123	emodin	Li et al. (2009)
		124	physcion	Zhao et al. (2008)
Esters		125	3-hydroxy-2-butanone	Za et al. (2008)
		126	ethyl acetate	Lin et al. (2000)
		127	1,2-Benzenedicarboxylic acid	Liu et al. (2004b)
		128	Ethyl palmitate	Liu et al. (2004a)
		129	(2*R*) 2-*O*-*β*-D-glucopyranose 2-phenylacetic acid methyl ester	Chen (2020)
		130	diisobutyl phthalate	Lao et al. (2018)

In addition to its use as a traditional Chinese medicine (TCM), the fruit of *Ficus hirta* Vahl is gaining traction in the food industry. The roots of *Ficus hirta* Vahl can be used to make tea bags, medicinal wine, functional drinks, ingredients, or drugs (Lin et al., 2013; Bhambhani et al., 2021). In recent years, to better utilize *Ficus hirta* Vahl as a resource, effectively promote the study of its pharmacological effects, and explore its clinical applications, there has been a significant investment in research on *Ficus hirta* Vahl However, despite progress in this field, there has been no comprehensive and systematic overview of the current research findings. Therefore, this study aimed to comprehensively summarize the latest overview of the plant characteristics, ethnopharmacology, phytochemistry, pharmacology, different potential uses, and toxicity of *Ficus hirta* Vahl to lay the foundation for further studies on this botanical drug, as well as to accelerate the application of *Ficus hirta* Vahl in the sustainable development of future agriculture, food, and pharmaceutical industries.

## 2 Methods

The available information on *Ficus hirta* Vahl was collected from scientific databases published from 1993 up to 2025. Information on *Ficus hirta* Vahl was obtained from published sources, including monographs on medicinal plants, ancient and modern recorded classics, the Chinese Pharmacopoeia and electronic databases, such as Web of Science, PubMed, CNKI, Wanfang DATA, Google Scholar, Baidu Scholar and Flora of China (FOR). The search terms used for this review included “*Ficus hirta* Vahl,” “*wuzhimaotao*,” “*Ficus hirta* Vahl,” “*tuhaungqi*,” “*radix fici simplicissimae*,” “*Fici Hirtae Radix*,” “*Hairy Fig*,” and “*wuzhualong*” all of which are accepted names and synonyms, “botanical characterization,” “compounds,” “traditional uses,” “pharmacology,” “toxicology,” “quality standard,” “extraction and purification” and “applications”. No language restrictions were applied during the search.

## 3 Botanical characterization and distribution of *Ficus hirta* Vahl

**FIGURE 1:**
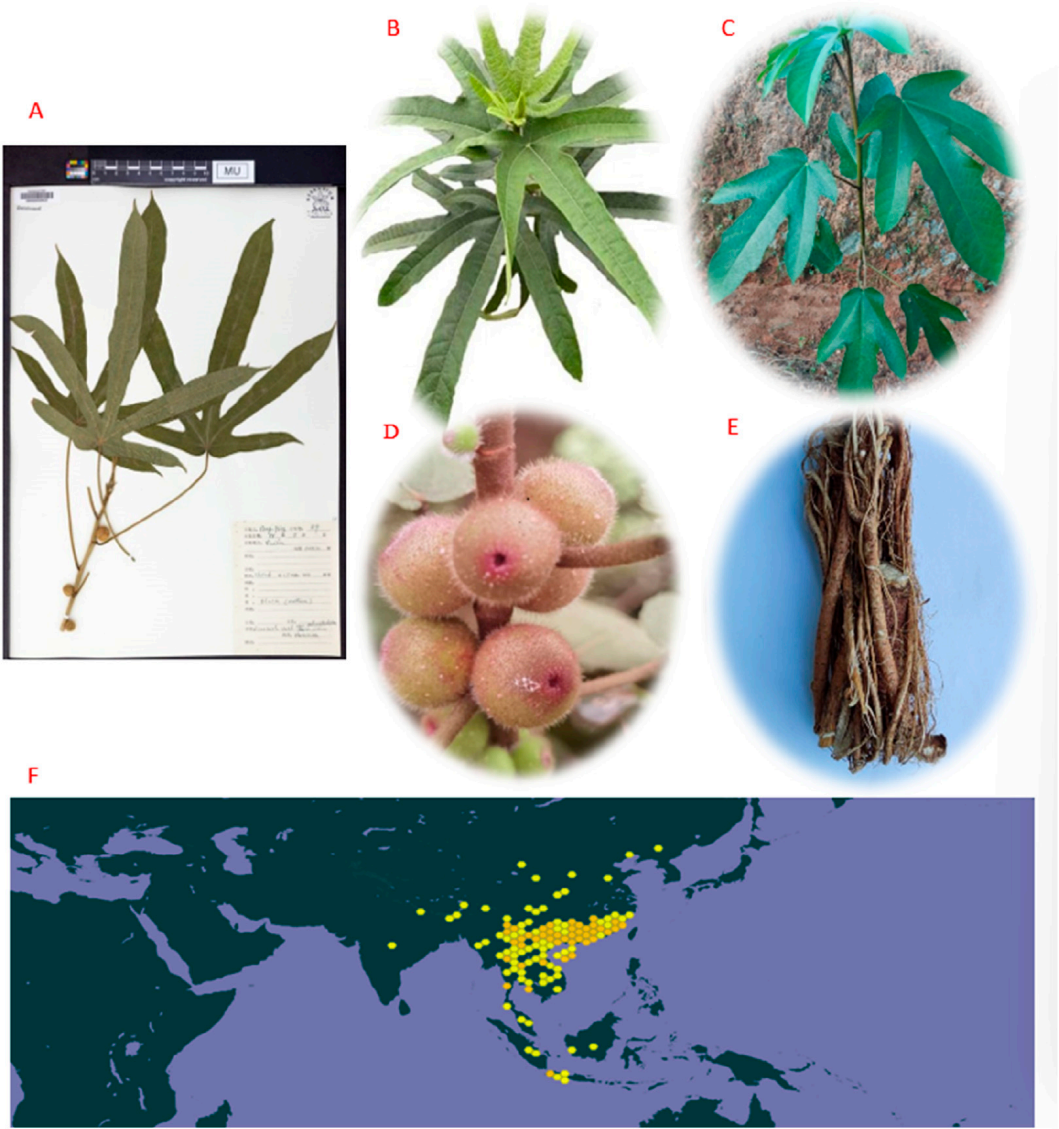
Plant specimen **(A)** (https://www.gbif.org/occurrence/4850269523), leafs **(B)**, stem **(C)**, fruits **(D)**, roots **(E)**, and the world distribution map **(F)** (https://www.gbif.org/species/8213018; https://image.baidu.com) of *Ficus hirta* Vahl.

Interestingly, the name “*Wuzhi Maotao*” is derived from the plant’s characteristics, as it typically has five-lobed leaves shaped like five fingers, which is described as “*Wuzhi*.” *Ficus hirta* Vahl is also known as *Niunai Mu* or *Wuzhua Long*. The fruit of *Ficus hirta* Vahl resembles a peach and is covered with fluff. The leaves are alternate, oblong, lanceolate, or narrowly ovate, with microwave-like serrations. Most leaves have five lobes, yet some may have one, three, or seven (Yu et al., 2018). *Ficus hirta* Vahl often matures in autumn and flowers in the summer. The crisp and tasty fruit develops from the bottom to the top, growing in pairs with branches (Tian et al., 2023). Plants also produce milky juice. It is a common plant that grows in valleys, woodlands, and at sea level on slopes above 500–1000 m, and it is an annual botanical reaching a height of 1–2 m (Zhou, 2013). *Ficus hirta* Vahl is widespread in Southeast Asian countries, such as Nepal, Bhutan, northeastern India, Vietnam, Myanmar, Thailand, Malaysia, Cambodia, and Indonesia. In China, *Ficus hirta* Vahl is mainly distributed in Yunnan, Guizhou, Guangxi, Canton, Hainan, Hunan, Fujian, Jiangxi, and other regions. It is ranked among the ten most famous botanical drugs in Southeast China (Zhang and Du, 2021a). Studies have shown that *Ficus hirta* Vahl has various pharmacological effects, abundant metabolites, rich nutritional value, and a strong application potential (Gu et al., 2022; Huang et al., 2020). Plant specimen (A https://www.gbif.org/occurrence/4850269523), leafs (B), stem (C), fruits (D), roots(E) and the world distribution map (F) of *Ficus hirta* Vahl in Figures 1A–F.

## 4 Traditional use

Medicinal records of *Ficus hirta* Vahl first appeared during the Qing Dynasty and have been continuously enriched in subsequent generations. The medicinal properties and clinical applications of *Ficus hirta* Vahl recorded in ancient times are consistent with those reported in literature (Priya et al., 2015). According to TCM theory, the taste of *Ficus hirta* Vahl is sweet and when taken as a medicine, it is distributed to the lung, spleen, and liver meridians (Luo, 2009). Additionally, *Ficus hirta* Vahl is a multi-ethnic Chinese herbal medicine, which was recorded in Yao, Zhuang, Dai, Dong, Li, Hani, and other ethnic medicine books. These books record the medicinal value, original plants, and geographical distribution of *Ficus hirta* Vahl (Shi et al., 2018).

During the Qing Dynasty, *Ficus hirta* Vahl was first documented in the *Shengcao Yaoxing Beiyao.* It has been used to treat scabies, moss, and hemorrhoids. It is also used to wash hemorrhoids and relieve pain and swelling in the skin. *Zhiwu Mingshi Tukao* found that *Ficus hirta* Vahl is effective in treating arthritis and swelling. The records of *Lingnan Caiyaolu* in 1932 on its medicinal efficacy were similar to that reported previously. According to *Zhongyao Zhi* (1959), this plant is used in traditional medicine to stimulate the spleen, tonify the lungs, remove moisture, and soothe tendons (Zhou, 2013). It adds the effect of energizing the spleen, tonifying the lung, and it is often applied in gynecological diseases. The descriptions of *Ficus hirta* Vahl in the four ancient medicinal books from *Guangxi folk commonly used herbal manual*, *New Medical Newslette*r, *Anthology of single prescription of Hunan Chinese herbal medicine*, and *National C. herbal medicine compilation* are relatively brief (Zhang and Qu, 2021). The clinical applications reported in *Practical C. herbal medicine* include treating stomach pain, bronchitis, and mastitis. In 1977, *Pharmacopoeia of the People’s Republic of China (Volume I)* documented its use in the treatment of dropsy, rheumatic arthralgia, and lower back pain. This indicates that *Ficus hirta* Vahl has a statutory standard, but its records are relatively brief. In 1984, *Zhongguo Minzu Yaozhi* reported that *Ficus hirta* Vahl can be used to treat indigestion, abdominal distension, abdominal pain, cough, neurasthenia, asthma, chronic hepatitis, and maternal hypolactation (Luo, 2009). In 1999, *Zhonghua Bencao* expanded on the clinical applications and efficacy of *Ficus hirta* Vahl No new clinical applications have been suggested in the *Canton Provincial Standards of Chinese Medicinal Materials*. In 2013, *Ficus hirta* Vahl was included in the *Guangxi Zhuang Autonomous Region Yao medicine quality standard (Volume I)* as a *Yao* medicine. In 2015, the *Chinese Pharmacopoeia (Volume I)* recorded *Ficus hirta* Vahl as a medicinal material for the prescription of Chinese patent medicines (Zhong and Xu, 2000; Wu and Zhou, 2023). Not only is the root used medicinally but recent literature on *Ficus hirta* Vahl also lists the fruit as a medicinal component (Chen et al., 2024). Nevertheless, there are few clinical studies on the fruit and other plant parts such as leaves and stems, and the use of these parts are rarely supported by the literature, warranting further discussion and study. To optimize the benefits of *Ficus hirta* Vahl, the mixed use of medicinal and non-medicinal parts should be rigorously controlled because of variations in the effective components of the different parts (Vu et al., 2023).

Overall, traditional application records indicate that *Ficus hirta* Vahl can be widely used to treat indigestion, loss of appetite, cold, pneumonia, tuberculosis, cough, asthma, night sweats, low back pain, bruises, arthritis, liver disease, skin diseases, gynecological diseases, and pediatric diseases, with almost no adverse reactions. *Ficus hirta* Vahl is particularly beneficial for treating gastrointestinal issues. According to TCM, the therapeutic benefits of *Ficus hirta* Vahl in strengthening the lungs and spleen, promoting qi, and removing dampness are important focal points. A summary of the traditional uses of *Ficus hirta* Vahl is presented in Table 2.

**TABLE 2:** A summary of the traditional uses of *Ficus hirta* Vahl is shown in table.

The title of the books	Period	Medicinal parts	Traditional use	References
*Shengcao Yaoxing Beiyao*	Qing dynasty	Root	Scabies moss, hemorrhoids, swollen skin pain, cough, bruises, and injuries from falls, rheumatism	Zhou (2013)
*Zhiwu Mingshi Tukao*	Qing dynasty	Root	Arthritis and swelling	Zhou (2013)
*Lingnan Caiyaolu*	1932	Root	Scabies moss, hemorrhoids, swollen skin pain, cough, bruises, and injuries from falls, rheumatism	Zhou (2013)
*Zhongyao Zhi*	1959	Root	Loss of appetite, fatigue, consumption cough, night sweats, rheumatism, hepatitis, leukorrhea, *postpartum* agalactia, eczema	Zhang and Du (2021b)
*Guangxi folk commonly used herbal manual*	1969	Root	Carbuncle, sore, swollen skin pain, *postpartum* agalactia, abnormal leukorrhea	Zhang and Du (2021a)
*practical Chinese herbal medicine*	1970	Root, Fruit	Tuberculosis, cough, mastitis, chronic bronchitis, night sweats, orchitis, rheumatism, all kinds of nameless swelling, stomach pain, chest pain, chronic hepatosplenomegaly, eczema	Luo (2009)
*New Medical Newsletter*	1972	Root	Jaundice, hepatitis, eczema	Luo (2009)
*Anthology of single prescription of Hunan Chinese herbal medicine*	1973	Root	Treat unknown swelling poison rheumatism	Luo (2009)
*National Chinese herbal medicine compilation*	1975	Root	Tuberculosis, cough, chronic bronchitis, rheumatoid arthritis, back and leg pain, edema, sweating disease	Luo (2009)
*Pharmacopoeia of the People’s Republic of China*	1977	Root	Edema, sweating, rheumatism, arthralgia, and lumbago	Luo (2009)
*Zhongguo MinzuYaozhi*	1984	Root	Dyspepsia, abdominal distension, stomachache, cough, neurasthenia, asthma, chronic hepatitis, maternal lactation, hematuria, urine retention, proctocele, muscle and bone pain	Luo (2009)
*Zhonghua Bencao*	1999	Root	edema, fatigue, consumption, cough, sweating disease, abnormal leucorrhea, *postpartum* agalactia, rheumatism and arthralgia, edema, cirrhosis of the liver ascites, hepatitis, muscle and bone pain	Zhong and Xu (2000), Wu and Zhou (2023)
*Guangdong Provincial Standard of Chinese medicinal materials*	2004	Root	Phlegm, wheezing, spleen and stomach qi deficiency, limb fatigue, abdominal distension, edema, abnormal leucorrhea, rheumatism and arthralgia, waist and leg pain	Zhong and Xu (2000), Wu and Zhou (2023)
*Chinese medicine dictionary*	2006	Root	Cure tuberculosis cough, rheumatism arthralgia pain, cirrhosis of the liver ascites, hepatitis, *postpartum* agalactia, abnormal leucorrhea, night sweats	Zhong and Xu (2000), Wu and Zhou (2023)
*Hunan provincialstandard of Chinese medicinal materials*	2009	Root	Phlegm, wheezing, Qi deficiency, limb fatigue, abdominal distension, edema, abnormal leucorrhea, rheumatism and arthralgia, waist and leg pain	Zhong and Xu (2000), Wu and Zhou (2023)
*Guangxi Zhuang Autonomous Region Yao medicine quality standard (Volume I)*	2014	Root	Qi deficiency, pulmonary nodules, tracheitis, hepatitis, cirrhosis of the liver ascites, stomach pain, abdominal pain, rheumatic heart disease, anemia, *postpartum* weakness	Zhong and Xu (2000), Wu and Zhou (2023)

## 5 Phytochemistry

### 5.1 Flavonoids

Flavonoids have attracted attention in the field of drug research and development owing to their multiple biological activities and complex mechanisms of action (Wang et al., 2019; Panchal et al., 2021). Flavonoids are the principal active metabolites in *Ficus hirta* Vahl They play an essential role in the growth and development of plants, enhance plant resistance to unfavorable environmental conditions, and promote human wellbeing. Additionally, certain flavonoids can alter the coloration of flowers and fruits, while enhancing the flavor of food (Li et al., 2014; Sun et al., 2013). They have a general 2-phenylchromone structure (Liu and Xue, 2016). Flavonoids derived from *Ficus hirta* Vahl exhibit a wide range of biological activities, including, among others, anti-inflammatory, antibacterial, antioxidant, anti-aging, antiviral, and anticancer effects (Wen et al., 2021). Two novel flavonoids, naringenin-7-O-β-D-glucoside and pinocembrin-7-O-β-D-glucoside were isolated from *Ficus hirta* Vahl and their structures were elucidated using spectroscopic methods, including nuclear magnetic resonance (NMR) spectroscopy and electrospray ionization mass spectrometry (ESI-MS). These flavonoids have remarkable anti-cancer and free radical-scavenging properties (Wan et al., 2017). Four common flavonoids (tricin, quercetin, vitexin, and cyclomorusin) were identified using a general method, and quercetin was found to possess significant anti-inflammatory properties (Zheng et al., 2013a). Flavonoids in *Ficus hirta* Vahl seedlings mainly accumulate in the leaves and stems (Chen et al., 2021a). In another study, the roots and leaves of *Ficus hirta* Vahl had higher flavonoid pigment extract contents than other plant parts (Liu et al., 2023). These two experiments showed that the flavonoid content in *Ficus hirta* Vahl was closely related to the planting environment and planting time; therefore, the flavonoid content may vary with different planting areas and increase with extended planting time. Each 100 g of *Ficus hirta* Vahl contains approximately 351.68–352.18 mg of flavonoids, which supports the subsequent development and utilization of the plant (Tang et al., 2021).

**FIGURE 2:**
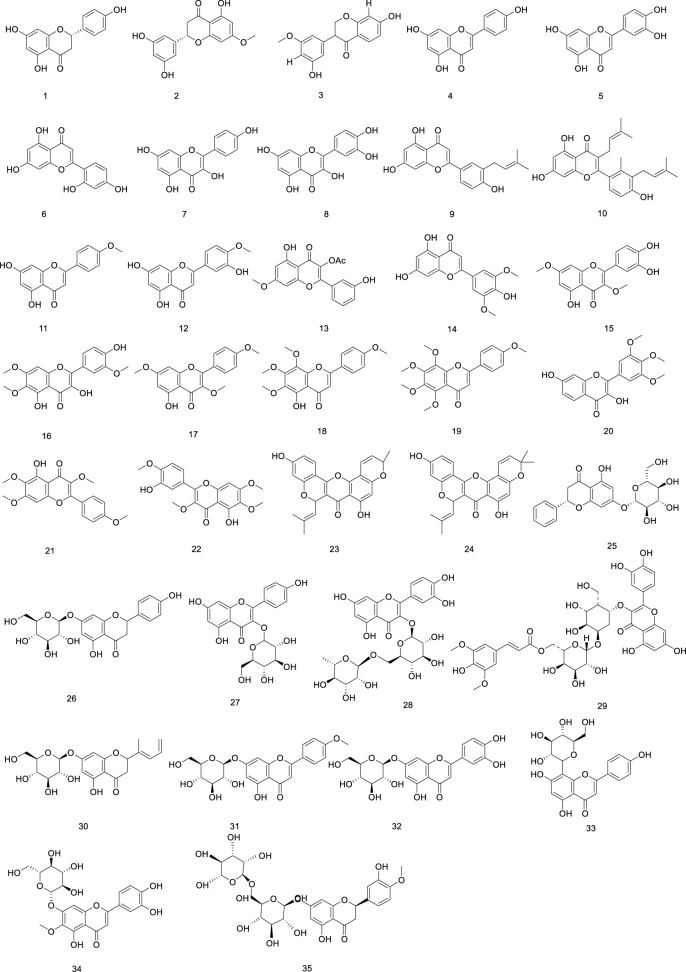
Chemical structure of flavonoids·(compounds·1–35) in *Ficus hirta* Vahl

To date, a total of 34 flavonoids have been isolated from *Ficus hirta* Vahl Apigenin, luteolin, kaempferol, quercetin, and rutin were identified and quantified. Although many flavonoids have been discovered and isolated from *Ficus hirta* Vahl, detailed pharmacological studies of each flavonoid have not yet been conducted (Cheng et al., 2017). Additionally, some flavonoids from *Ficus hirta* Vahl lack distinctive biological actions, and we anticipate the isolation of new flavonoids from *Ficus hirta* Vahl The flavonoids (compounds 1–35) isolated from *Ficus hirta* Vahl are presented in Table 1; Figure 2.

### 5.2 Phenylpropanoids

**FIGURE 3:**
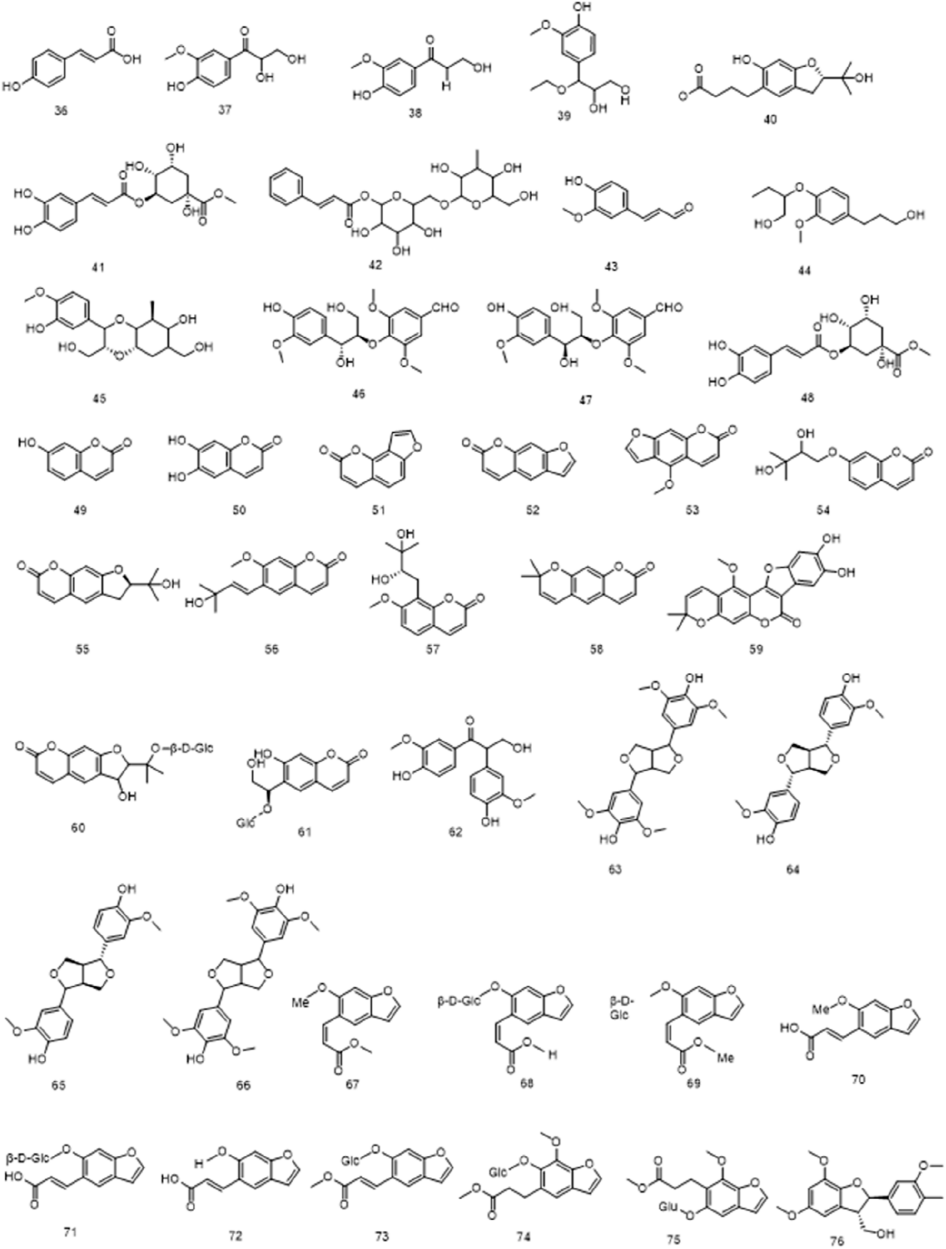
Chemical structure of phenylpropanoids·(compounds·36–76) in *Ficus hirta* Vahl

Phenylpropanoid metabolites in *Ficus hirta* Vahl can be divided into several categories, including simple phenylpropanoids (36–48), coumarins (49–61), simple lignans (62), bisepoxylignans (63–66), and benzofuran lignans (67–76). Among these, coumarin metabolites represent an important type of naturally occurring and synthetic oxygen-containing heterocyclic compounds with a typical benzopyrone framework. Chemical modifications of coumarins have been shown to improve their antibacterial activity (Annunziata et al., 2020; Tu et al., 2021). Coumarin metabolites exhibit various biological properties, including anti-inflammatory, anti-cancer, and hepatoprotective effects. They can also be used to treat metabolic diseases (Bhattarai et al., 2021). The structure of (E)-suberenol was established based on extensive spectroscopic analyses, including NMR spectroscopy and high-resolution (HR)ESI-MS. Additionally, meranzin hydrate has been discovered in *Ficus hirta* Vahl using MS and infrared (IR), ultraviolet (UV), and NMR spectroscopy (Zheng et al., 2013b). Both coumarins exhibit remarkable antioxidant and anti-inflammation properties (Ya et al., 2010; Kang et al., 2020). A previous study revealed that psoralen is the main metabolite of the total coumarins in *Ficus hirta* Vahl, and pharmacological studies have shown that psoralen has strong antibacterial, anticancer, anti-osteoporosis, and neuroprotective activities (Tao et al., 2011; Zhong et al., 1993). In recent years, coumarins have been suggested to have high activity and toxicity, some of which include hepatotoxicity and nephrotoxicity (Garg et al., 2020; Srikrishna et al., 2018). Overall, although many coumarins have been discovered and isolated from *Ficus hirta* Vahl, detailed pharmacological studies of each coumarin have not yet been conducted. The stems, leaves, flowers, fruits, roots, and other components of *Ficus hirta* Vahl contain abundant simple lignans with a range of structural variants and biological roles. Plant lignans can only be converted into animal lignans in the microecological environment of the gastrointestinal tract (Nawfetrias et al., 2024). Lignans play an important role in regulating estrogen levels, and neurodegenerative diseases. They also exhibit antitumor effects (Li et al., 2023a; Wang et al., 2022a). Lignans are powerful weapons used by plants to resist stress and have diverse bioactive functions that protect human health (Li et al., 2023b). Lignans are plant-active metabolites with estrogen- or anti-estrogen-like activities that can bind to estrogen receptors (ER) and play an estrogen-like role; therefore, they are classified as phytoestrogens. Clinical applications of *Ficus hirta* Vahl therefore include stimulating mammary gland milk secretion, improving *postpartum* mammary gland milk secretion, and improving the quality and quantity of breast milk (Peñalvo et al., 2008). However, these metabolites are present in other plant species and are not unique to *Ficus hirta* Vahl The phenylpropanoids (compounds 36–76) isolated from *Ficus hirta* Vahl are presented in Table 1; Figure 3.

### 5.3 Phenolics and phenolic glycosides

**FIGURE 4:**
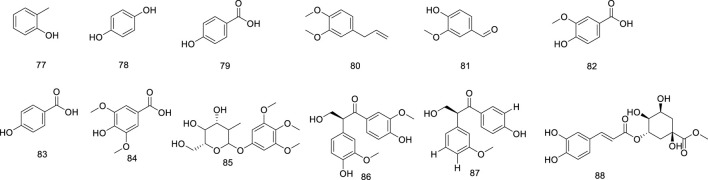
Chemical structure of phenolics·(compounds·77–88) in *Ficus hirta* Vahl

**FIGURE 5:**
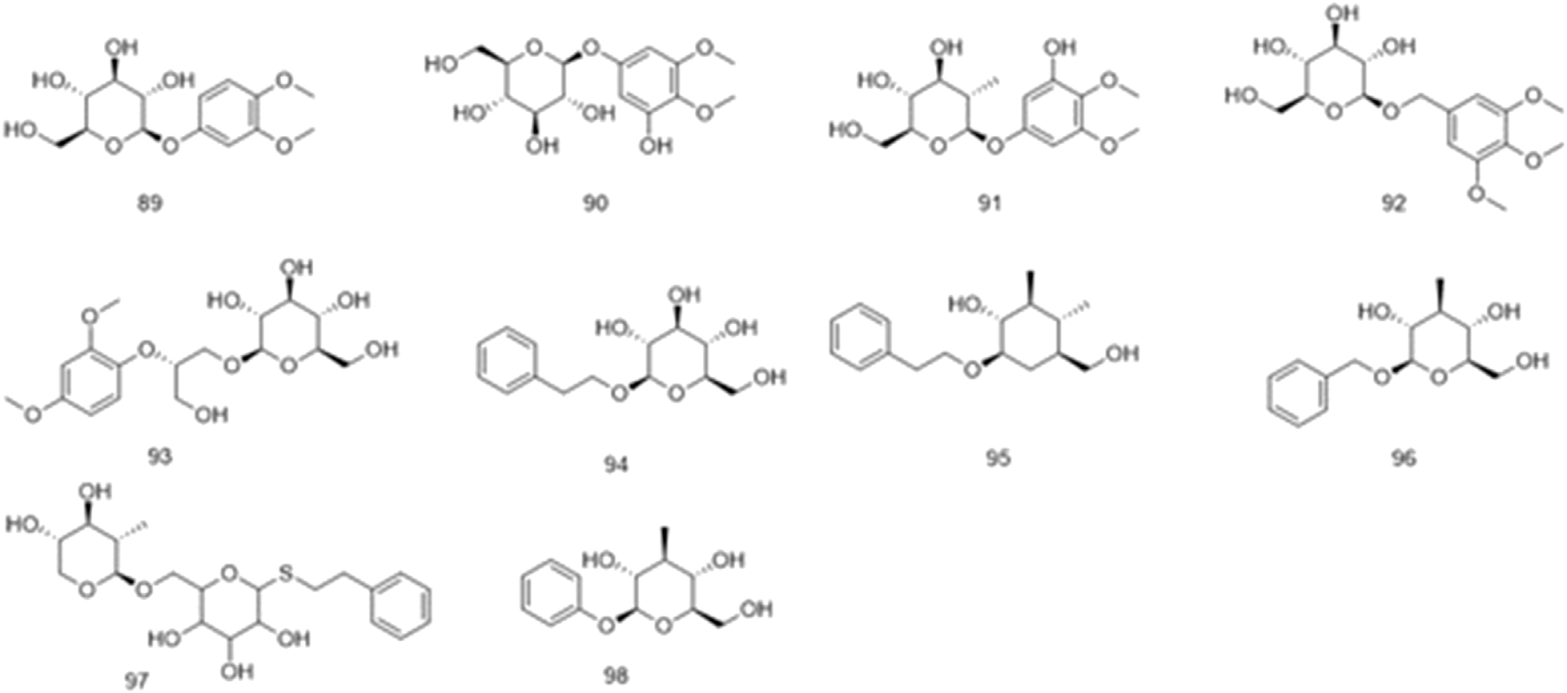
Chemical structure of phenol glycosides·(compounds·89–98) in *Ficus hirta* Vahl

Phenolics are ubiquitous in the plant kingdom and are distributed in the roots, stems, leaves, and fruits of plants (Amadi et al., 2022; Ou et al., 2019). Phenolic substances are organic metabolites with a benzene ring structure, most of which have an aroma (Alara et al., 2021). The phenolic metabolites of *Ficus hirta* Vahl exert anticancer effects (Yi et al., 2019). HRESI-MS, HPLC techniques, and NMR, IR, and UV spectroscopy were used to detect vanillin, vanillic acid, p-hydroxybenzoic acid, syringic acid, ficuglucoside, and evofolin B. (8R)-4, 5′ dihydroxy-8 hydroxymethyl-3′-methoxy deoxybenzoin, methyl chlorogenic acid, hydroquinone and cresol were further obtained by ultrasonic-assisted extraction of the active metabolites in *Ficus hirta* Vahl and determination using high-performance liquid chromatography-mass spectrometry (HPLC-MS/MS). In the experiment, evofolin B showed a strong inhibitory effect on the lipopolysaccharide (LPS)-induced release of nitric oxide (NO) from Raw 264.7 (IC_50_ 10.10–59.86 μmol/L) and exhibited potent anti-inflammatory activity (Chen, 2017). Spectroscopic techniques such as IR, UV, NMR, and ESI-MS have been used to isolate methyleugenol from *Ficus hirta* Vahl and identify its structure (Zheng et al., 2013a). Phenolic glycosides are formed by condensation of the phenol hydroxyl group in the aglycone molecule and the end-group carbon atom of the sugar (Guo et al., 2014). Phenolic glycosides, constituents of *Ficus hirta* Vahl, are natural metabolites ubiquitously found in various food sources, such as fruits, vegetables, and Chinese medicinal botanical drugs (Murugesu et al., 2021). Some well-known Chinese botanical drugs, such as *Leonurus japonicus* Houtt and *Curculigo orchioides* Gaertn, are rich in phenolic glycosides and exhibit anti-inflammatory activities (Liu et al., 2022). Phenolic glycosides decrease neuroinflammation in BV2 microglia cells, which might be regulated by inhibiting protein activity in the NF-κB and MAPK (JNK and ERK1/2) signaling pathways. Thus, there is a good prospect that it can be utilized to treat neurodegenerative diseases (Ye et al., 2020). It possesses antioxidant, anti-cancer, and immunomodulatory properties (Conforti and Menichini, 2011). Phenolic glycosides in *Ficus hirta* Vahl are not only used in the drug development but also in food production; they can enhance the taste of food and have anti-corrosive and preservative effects (Sun and Shahrajabian, 2023; Lu et al., 2006). The phenolics (compounds 77–88) phenol glycosides (compounds 89–98) isolated from *Ficus hirta* Vahl are presented in Table 1; Figures 4, 5.

### 5.4 Terpenoids

**FIGURE 6:**
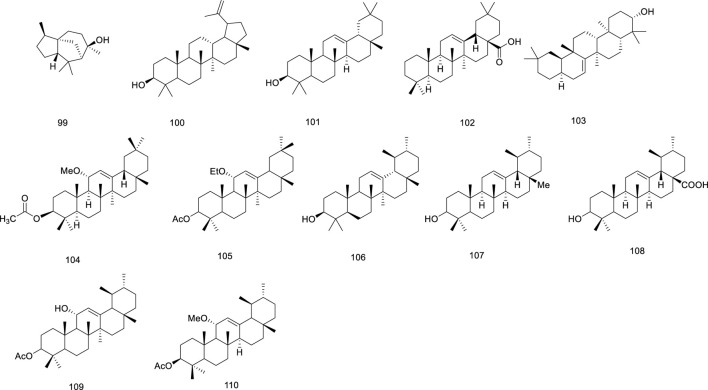
Chemical structure of terpenoids·(compounds·99–110) in *Ficus hirta* Vahl

Terpenoids are important secondary metabolites in medicinal plants and triterpenoids are the most commonly extracted components from *Ficus hirta* Vahl (Bergman et al., 2019; Tholl, 2015). Terpenoids are vital organic molecules present in plants and are considered to have a variety of biological activities, including anti-inflammatory, antibacterial, antiviral, antitumor, antioxidant, and cardioprotective effects (Li et al., 2023c). The terpenoids (compounds 99–110) isolated from *Ficus hirta* Vahl are presented in Table 1; Figure 6.

### 5.5 Sterols

**FIGURE 7:**
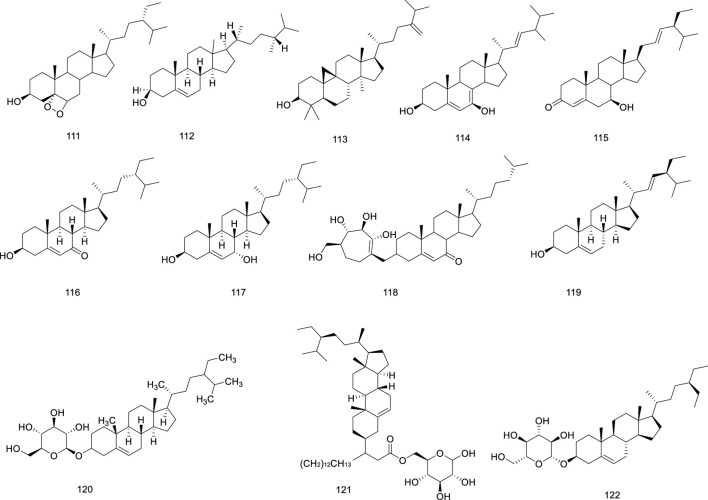
Chemical structure of sterols·(compounds·111–122) in *Ficus hirta* Vahl

Twelve sterols have been identified in *Ficus hirta* Vahl Sterols are structural components of *Ficus hirta* Vahl plant membranes that regulate membrane fluidity and permeability to molecules such as proteins (Ferrer et al., 2017). Sterols play an important role in the growth and development of organisms and are precursors of various steroid hormones in eukaryotes; therefore, they are also called sterols (Evtyugin et al., 2023). Sterols perform a wide range of biological functions, including anti-oxidant, anti-cancer, and blood lipid level reducing activities (Couder-García et al., 2019; Cusack et al., 2013). The structure of β-daucosterol isolated from *Ficus hirta* Vahl was elucidated using UV and NMR spectroscopy and ESI-MS (Lao et al., 2018). The sterols (compounds 111–122) isolated from *Ficus hirta* Vahl are presented in Table 1; Figure 7.

### 5.6 Quinones

**FIGURE 8:**
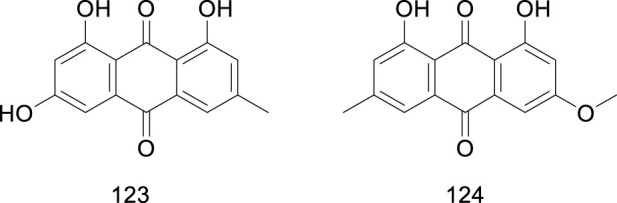
Chemical structure of quinones·(compounds·123–124) in *Ficus hirta* Vahl

Quinones are a family of natural and synthetic metabolites characterized by a fully conjugated cyclic unsaturated dione structure. Emodin (1,3,8-trihydroxy-6-methyl-anthraquinone) is a natural anthraquinone derivative present in numerous globally renowned herbal medicines. Studies have shown that quinones display diverse biological activities, including neuroprotective, antitumor, and renoprotective effects (Li, 2019; Zhao et al., 2023). Emodin was isolated in *Ficus hirta* Vahl (Li et al., 2009); interestingly, emodin was found to have diuretic and purgative effects, echoing the traditional use of *Ficus hirta* Vahl in dehumidifying and reducing swelling. In addition to emodin, physcion ether has been isolated from *Ficus hirta* Vahl (Zhao et al., 2008). Physcion ether plant fungicides can not only directly inhibit the growth of plant pathogens, but also inhibit the activity of citrus postharvest pathogens and activate the active immune system of plants. The quinones (compounds 123–124) isolated from *Ficus hirta* Vahl are presented in Table 1; Figure 8.

### 5.7 Esters

**FIGURE 9:**
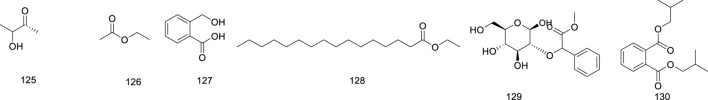
Chemical structure of esters·(compounds·125–130) in *Ficus hirta* Vahl

Esters, which make up vegetable oils and contribute to the fragrant flavor of plants, are essential plant components (Derbassi et al., 2022). Esters are a class of metabolites formed when acids and alcohols are dehydrated (Saini et al., 2024). Esters have been known to be applicable in medicine, food, and cosmetics (Chen, 2020; Wang et al., 2022b). The volatile composition of *Ficus hirta* Vahl was found to contain 7%–15% ethyl acetate, which was obtained by steam distillation (Lin et al., 2000). The Esters (compounds 125–130) isolated from *Ficus hirta* Vahl are presented in Table 1; Figure 9.

### 5.8 Other metabolites

Eight aldehydes have been isolated from *Ficus hirta* Vahl, namely, hexanal, heptanal, octanal, nonanal, (2E)-2-octenal, 1-decanal, (2E)-2-nonenal, and 2-butyloct-2-enal (Li et al., 2010). These chemicals were identified using GC-MS. It was found that the aldehydes changed significantly with an increase in the drying temperature. The higher the hot-air drying temperature, the higher was the relative content of aldehydes, with aldehyde contents of 6.86% at 40°C and 11.02% at 70°C (Gui et al., 2021). Both plants and humans require mineral elements for their healthy growth and development. Atomic absorption spectroscopy was used to determine the mineral contents which were present in the following order: Ca > Mg > Mn > Fe > Cu > Pb (Wen et al., 2010). The aboveground parts of *Ficus hirta* Vahl were rich in mineral elements. The mineral content in leaves and fruits was higher than that in stems. Intercropping under rubber plantations increased the contents of N, K, Mg, Mn, Cu, and Zn and reduced the contents of Ca and Fe (Yuan et al., 2023).

## 6 Pharmacological effects and therapeutic potential of *Ficus hirta* Vahl

**FIGURE 10:**
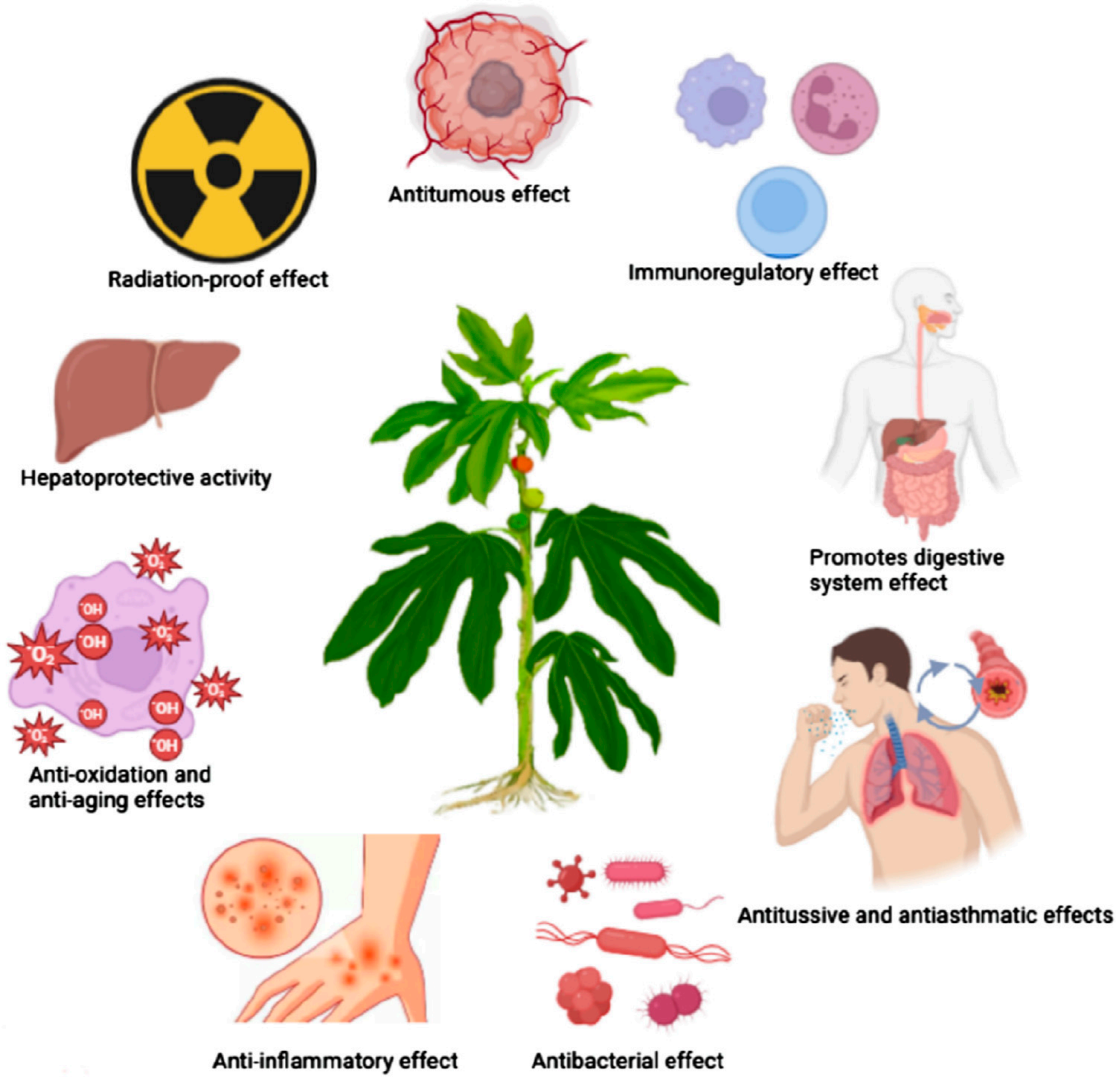
Pharmacological effects of *Ficus hirta* Vahl (created by Figdraw: https://www.figdraw.com).

Pharmacological studies have shown that *Ficus hirta* Vahl has immunoregulatory, digestive system function promoting, antitussive, antiasthmatic, antibacterial, anti-inflammatory, antioxidant, anti-aging, hepatoprotective, anti-radiation, and antitumor effects. The pharmacological effects target of *Ficus hirta* Vahl is summarized in Figure 10. Pharmacological studies of *Ficus hirta* Vahl in Table 3.

**TABLE 3:** Pharmacological studies of *Ficus hirta* Vahlis shown in table.

Effect	Part of plant/Extracts or compound	Model	positive control	Formulation on/dosage	Effective dose	MIC/MFC/IC_50_	Result	References
Immunoregulatory effect	Radix/aqueous	cyclophosphamide (40 mg/kg)	gynostemma total glycoside tablets (20 mg/kg)	*in vivo* 2 g/kg 4 g/kg 6 g/kg	carbon particle clearance function: 4 g/kg Serum hemolysin levels and immune organ weight index 2 g/kg 4 g/kg 6 g/kg	—	The study found that Ficus hirta significantly enhanced the immune function of mice with compromised immune systems by increasing the clearance rate of carbon particles, the weight indices of the thymus and spleen, and the level of hemolysin in the serum	Liu et al. (2004b)
	Radix/aqueous	cyclophosphamide (50 mg/kg)	—	*in vivo* 2.2 g/kg 4.4 g/kg 6.6 g/kg	4.4 g/kg 6.6 g/kg	—	The study found that aqueous extracts of Ficus hirta significantly enhanced cellular immunity in immunosuppressed mice by increasing the spleen index, macrophage phagocytosis, T cell proliferation and killing activity, T lymphocyte subgroup quantity, and levels of IL-1β and INF-γ	Yang et al. (2015)
Promotes digestive system effect	Radix/aqueous	100% rhubarb soaked in decoction	atropine (20 m g/kg)	*in vivo* 6.67 g/kg 13.34 g/kg 26.67 g/kg	13.34 g/kg 26.67 g/kg	—	The aqueous extracts of Ficus hirta significantly reduced the gastric emptying rate and small intestine propulsion rate in a rat model of spleen deficiency (P < 0.05–0.01), and inhibited the motility of the small intestine (P < 0.05). The extracts alleviated the excessive small intestine propulsion rate and improved spleen deficiency symptoms in the rat model	Luo et al. (2012a)
	Radix/aqueous	ligation of the pylorus of rats	igustrazine (25 mg/kg)	*in vivo* (Rats) 2.5 g/kg 5 g/kg 10 g/kg *in vivo* (Mice):t 5 g/kg 10 g/kg 20 g/kg	10 g/kg, 20 g/kg	-	The aqueous extract of Ficus hirta protects the gastric mucosa and improves microcirculation, with a trend of reducing gastric acid and pepsin secretion in pyloric-ligated rats, and significantly enhances ear microcirculation in mice	Wang et al. (2011a)
	Radix/aqueous	indomethacin (40 mg/kg)	—	*in vivo* 7 g/kg 14 g/kg	14 g/kg	—	Improve the expression of gastric mucosal tight junction protein (ZO-1, Claudin-1, Occludin) and adherent junction protein (E-cadherin, α-catenin) It had an ameliorating effect on the expression of SOX2 in gastric mucosa, but had no significant effect on the expression of CDX2	Zeng et al. (2020)
	Radix/aqueous	reserpine (1 mg/kg)	—	*in vivo* 5 g/kg 10 g/kg 20 g/kg	20 g/kg	—	Increase the concentration of β-EP, MTL, and GAS in the plasma	Yang and Xia (2012)
Antitussive and antiasthmatic effects	Radix/aqueous	—	methorphan: 10 mg/kg aminophylline: 1200 mg/kg	*in vivo* (Rats) 5 g/kg 10 g/kg 20 g/kg *in vivo* (guinea pig) 4 g/kg 8 g/kg 16 g/kg	20 g/kg 16 g/kg	—	FhV reduced cough frequency and prolonged cough latency in mice and guinea pigs. It also inhibited asthma caused by histamine and acetylcholine, antagonized tracheal smooth muscle contraction caused by histamine phosphate, and had an anti-anaphylactic shock effect in guinea pigs	Zhou et al. (2009b)
	Radix/ethanol	—	—	*in vivo* (Rats) 8 g/kg *in vivo* (guinea pig) 8 g/kg *in vivo* (frog) 2 g/mL *in vitro* 500 mg/mL	8 g/kg, 8 g/kg 2 g/mL 500 mg/mL	—	The extract increased the cough threshold in guinea pigs, boosted phenol red excretion in mice, sped up cilia movement in frogs, extended the asthma latent period in guinea pigs, and expanded the trachea volume *in vitro*	Zeng et al. (2002)
Antibacterial effect	Radix/aqueous	—	—	*in vitro*:/	1 g/mL	1 g/mL	The extract strongly inhibited *Staphylococcus aureus*, *Escherichia coli*, and *Bacillus subtilis* at 1 g/mL, but not Aspergillus niger and Aspergillus flavus	Wang et al. (2010a)
	Radix/aqueous	—	—	*in vitro*:/	0.025 g/mL 0.05 g/mL, 0.011.21 g/mL, 0.014.07 g/mL	0.025 g/mL 0.05 g/mL, 0.011.21 g/mL, 0.014.07 g/mL	The extract inhibited *Escherichia coli* and *Staphylococcus* *aureus* with minimal inhibitory concentrations of 11.21 and 14.07 mg/mL, respectively. The chromotest microassay was more sensitive than traditional methods, especially for deeply colored plant extracts	Chen and Ye (2012)
	Radix, Fruit, stems, leaves/pinocembrin-7-O-β-D-glucoside	penicillium italicum penicillium digitatum	—	*in vivo* 1 g/L 2 g/L 4 g/L 8 g/L *in vitro*:/	200 mL/L 800 mg/L 4000 mg/L	200 mg/L 800 mg/L 4000 mg/L	The ethanol extract of Ficus hirta Vahl. fruits effectively inhibited the growth of Penicillium italicum and Penicillium digitatum, common citrus fruit pathogens causing blue and green molds. Three flavonoid glycosides, including pinocembrin-7-glucoside (P7G), were identified as key antibacterial compounds. P7G showed strong antifungal activity by inhibiting mycelial growth, spore germination, and disrupting the cell membrane and cell wall of P. italicum. The extract and its constituents also reduced postharvest blue mold in “Newhall” navel oranges by inhibiting P. italicum development	(Chen et al., 2020a Chen et al., 2020b Xia et al., 2016; Wan et al., 2017)
	Radix/ethanol	LPS(0.1,1,2,4 μg/mL)	—	*in vitro* 0 μg/mL, 2 μg/mL, 4 μg/mL, 8 μg/mL 10 μg/mL	4 μg/mL		FhV on LPS-induced apoptosis in orange-spotted grouper spleen cells. It finds that FhV alleviates LPS-induced apoptosis by activating miR-411, which inhibits Fas expression. This suggests that FhV could be a potential alternative to antibiotics for improving fish immunity and combating bacterial infections in aquaculture	Feng et al. (2024)
Anti-inflammatory effect	Radix/aqueous	xylene	—	*in vivo* 5 g/kg 10 g/kg 20 g/kg	20 g/kg	—	The extract significantly inhibits ear swelling caused by xylene and the increase in vascular permeability caused by acetic acid in mice	Zhou et al. (2008)
	Radix/ethanol	tail fin cutting	—	*in vivo* 5 mg/mL 10 mg/mL 20 mg/mL	5 mg/mL 10 mg/mL 20 mg/mL	—	Bergapten significantly inhibits the recruitment of neutrophils and macrophages to the injury site and promotes their clearance from the wound site. Additionally, bergapten effectively reduces the production of reactive oxygen species (ROS) and nitric oxide (NO) in the zebrafish model	Yang et al. (2018)
Anti-oxidant and anti-aging effects	Radix/polysaccharides, flavonoids, polyphenols, and amino acids	—	—	*in vitro*:/	—	—	The extract of ethyl acetate has the highest content of total flavonoids, while the extract of methanol has the highest content of total phenols. The extracts of 60% ethanol, 95% ethanol, and distilled water, as well as the traditional decoction method, have DPPH free radical scavenging abilities greater than 15%	Kuang et al. (2015)
	Radix/ethanol	—	—	*in vitro*:/	—	2.23 mg/mL, 0.33 mg/mL	The optimized extraction conditions for total coumarins from Ficus hirta are 58% ethanol, a 34:1 liquid-solid ratio, and 70 min of ultrasonic extraction, yielding 3.869 mg/g. The extracted coumarins show significant antioxidant activity with IC50 values of 2.23 mg/mL for DPPH and 0.33 mg/mL for ABTS free radicals	Wang et al. (2023)
	Radix/aqueous	100% rhubarb aqueous decoction 1 mL	atropine	*in vivo* 50 g/d 100 g/d 200 g/d	200 g/d	—	Ficus hirta water extract can slow down the gastric emptying speed and small intestinal propulsion speed in rats with spleen deficiency, and has an inhibitory effect on small intestinal motility	Luo et al. (2012b)
	Radix/aqueous	AlCl_3_(200 mg/kg)	—	*in vivo* 9g/(kg·d) 18g/(kg·d) 36g/(kg·d)	18g/(kg·d) 36g/(kg·d)	—	Medium and high doses of Radix Fici Hirtae significantly enhance memory function and reduce brain MDA while increasing SOD in AD mice (P < 0.05–0.01)	Feng and Li (2015)
	Radix/aqueous and ethanol	D-galactose (120 mg/kg)	VitE (100 mg/g)	*in vivo* 10 g/kg 20 g/kg	20 g/kg	—	FhV significantly boosts thymus and spleen indices, enhances serum SOD, liver GSH-Px, and brain CAT activities, and reduces serum MDA in aging mice	Wang et al. (2018), Ye et al. (2017)
Hepatoprotective effect	Radix/aqueous	50% ethanol and 0.1% CCl_4_ oil solution	indomethacin (25 mg/kg) biphenyl diester (5 mg/kg)	*in vivo* 5 g/kg 10 g/kg 20 g/kg	20 g/kg	—	Radix Fici Hirtae significantly reduces serum transaminase levels in cocaine-intoxicated mice in a dose-dependent manner, and improves liver tissue pathology	Cai et al. (2007), Zhou et al. (2008)
	Radix/aqueous	restraint stress	Ganoderma lucidum spore powder (GL) group (500 mg/kg)	*in vivo* 125 mg/kg 250 mg/kg 500 mg/kg	500 mg/kg	—	The extracts significantly lower plasma ALT and liver MDA, NO, while boosting liver ORAC, GSH, GSH-Px, and GST levels	Wang et al. (2015)
	Radix/aqueous	50% ethanol and 0.1% CCl_4_ oil solution	—	*in vivo* 0.75 g/kg 1.5 g/kg	1.5 g/kg	—	FhV pretreatment reduced CCl_4_-induced liver damage, as shown by lower serum ALT and AST levels and improved liver tissue health. Metabolomic analysis found 20 key markers related to amino acid and fatty acid metabolism	Zhou et al. (2022)
	Radix/aqueous	58% ethanol(10 mL/kg)	Kudzu group (0.53 g/(kg·d))	*in vivo* 0.2g/(kg·d) 0.4g/(kg·d) 0.6g/(kg·d)	0.4g/(kg·d) 0.6g/(kg·d)	—	FhV increase antioxidant markers (GSH content and SOD activity) and reducing inflammatory factor levels (IL-6, IL-1β, TNF-α and NF-κB)	Zhang et al. (2021)
	Radix/petroleum ether, chloroform, ethyl acetate and n-butanol	50% ethanol(12 mL/kg)	maltose solution	*in vivo* 50 mg/kg 100 mg/kg 200 mg/kg	50 mg/kg 100 mg/kg 200 mg/kg	—	The n-butanol group was an exception; other groups showed reduced hepatic lipid changes and inflammatory infiltration, with normal liver sinusoids. Chloroform and ethyl acetate groups had significantly lower serum AST, ALT, AKP, and LDH levels than the model group	Feng et al. (2018)
	Radix/aqueous	CCl_4_, MCD	olive oil	*in vivo* 13 g/kg 26 g/kg *in vitro*:/	13 g/kg 26 g/kg 1400 μg/mL 700 μg/mL	—	FhV reduced liver fibrosis and inflammation in mice, likely by modulating the GSH/GPX4 pathway to induce hepatic stellate cell ferroptosis	Feng et al. (2024)
Radiation-proof effect	polysaccharide	γ rays, X-rays, carbon tetrachloride (CCl4), methionine and choline deficiency diet (MCD), etc	saline	*in vivo*:/ *in vitro*:/	10 mg/kg 20 mg/kg 40 mg/kg 1400 μg/mL 700 μg/mL	—	Polysaccharides boost antioxidant levels, cut normal cell death, and shield against radiation harm. SNP from Sipunculus nudus aids DNA and bone marrow repair in irradiated mice. LBP from Lycium barbarum lessens oxidative stress and controls apoptosis in bone marrow cells. NTHSP from Hohenbuehelia serotina clears ROS and lessens DNA fractures. These also make tumor cells more sensitive to radiation, boosting radiotherapy results	Zhang et al. (2024)
	Radix/aqueous	6 Gy of 60Coγ rays	saline	*in vivo* 5 g/kg 10 g/kg 20 g/kg	5 g/kg 10 g/kg 20 g/kg	—	The extract significantly reduces DNA damage, as indicated by lower Tail DNA% and Tail Moment values in treated mice compared to the irradiated control group	Wang et al. (2010b), Wang et al. (2011b)
Antitumous effect	Radix/aqueous	cyclophosphamide (40 mg/kg)	saline	*in vivo*:/ *in vitro*:/	100 mg/L 200 mg/L 400 mg/L 5 g/kg 10 g/kg 20 g/kg	—	FhV can significantly reduce the micronucleus formation rate in bone marrow polychromatic erythrocytes and testicular germ cells induced by cyclophosphamide, indicating its potential antimutagenic function	Yang et al. (2019), Cen et al. (2010)
	Radix/Lignans et al.	—	—	—	—	28.88 μM	Lignans et al. induce apoptosis in HepG2 cells through the JNK and p38MAPK signaling pathways, inducing HeLa cell apoptosis through MAPK and AKT signaling pathways	Ye et al. (2022)
	Radix/ethanol	—	0.1% DMSO	*in vitro*:/	0.5 mg/mL 1.0 mg/mL 1.5 mg/mL 2.0 mg/mL 2.5 mg/mL	1.91 mg/mL 1.96 mg/mL 1.23 mg/mL	CAE, EAE, and BAE reduce HeLa cell viability and induce apoptosis in a dose-dependent manner, with EAE notably decreasing the G1 population, showing potential as an anticancer agent	Zeng et al. (2012)
	Fruit/DMSO	—	—	*in vitro*:/	6.25 μg/mL, 12.5 μg/mL, 25 μg/mL, 50 μg/mL 100 μg/mL	92.6 μg/mL, 7.68 μg/mL, 25.54 μg/mL	Psoralen inhibits BGC-803 cell growth with IC50 values of 92.6 μg/mL at 24 h, 25.54 μg/mL at 48 h, and 7.68 μg/mL at 72 h, and induces apoptosis by increasing early apoptotic rates	Yan et al. (2015)
	Fruit	ADR	—	*in vitro* 2 μg/mL 4 μg/mL 8 μg/mL 12 μg/mL 16 μg/mL 20 μg/mL	2 μg/mL 4 μg/mL 8 μg/mL 12 μg/mL 16 μg/mL 20 μg/mL	8 μg/mL	Psoralen significantly reduces MDR by inhibiting the efflux function of P-glycoprotein (P-gp), increasing the intracellular accumulation of adriamycin and rhodamine 123, without affecting P-gp protein or MDR1 mRNA expression levels	Jiang et al. (2016)

Note:“—”indicates that the type and specification are not clearly defind. “d” means “day”.

### 6.1 Immunoregulatory effect

The immune system is the most important defense mechanism against pathogen invasion. Because many plants have immunomodulatory properties, they have been used to treat many illnesses. An increasing number of studies have demonstrated that *Ficus hirta* Vahl can strengthen non-specific and specific immunity, improve immune system control, and be used to treat a variety of illnesses, including bone marrow suppression anemia, hemorrhagic anemia, myasthenia gravis, and other diseases (Yang et al., 2015; Huang et al., 2020). *Ficus hirta* Vahl significantly increased the charcoal clearance coefficient in cyclophosphamide-induced immunosuppressed mice, increased the weight index of the thymus and spleen, and increased serum hemolysin levels. These results indicate that *Ficus hirta* Vahl has a regulatory effect on immune function (Liu et al., 2004a). Fifty mice were randomly divided into the normal saline control, cyclophosphamide model, and *Ficus hirta* Vahl aqueous extract (6.6, 4.4, and 2.2 g/kg) groups. An immunocompromised mouse model was established using intraperitoneal injection of cyclophosphamide. After continuous intragastric administration for 14 days, *Ficus hirta* Vahl aqueous extract treatment (6.6 and 4.4 g/kg) significantly improved the spleen index of cyclophosphamide immunosuppressed mice. The phagocytic function of macrophages and the proliferation ability and killing activity of T lymphocytes were also significantly enhanced. The number of T cell subpopulations and the of interleukin (IL)-1 and interferon-γ levels increased. This indicates that *Ficus hirta* Vahl aqueous extract improved immune function in immunosuppressed mice, and its mechanism is related to the regulation of cellular immunity (Yang et al., 2015). *Ficus hirta* Vahl aqueous and alcohol extracts also have anti-stress effects, regulate immune function regulation, and improve anemia (Zhou et al., 2009a). These results provide scientific justification for *Ficus hirta* Vahl’s traditional usage as an immunoregulatory botanical drug, and offer a foundation for the development of innovative immunoregulatory medicines.

### 6.2 Digestion-promoting effect

As a well-known traditional botanical drug in various Asian nations, *Ficus hirta* Vahl has demonstrated remarkable efficacy in treating digestive system diseases by promoting food digestion and regulating the spleen and gastrointestinal tract (Hou et al., 2016; Theobald, 2015; Zheng et al., 2013b). *Ficus hirta* Vahl is frequently used to treat digestive disorders. In one study, normal rats were used as a control group, while spleen-deficient model mice were orally administered 100% rhubarb water infusion decoction. The gastric emptying and intestinal propulsion speed of mice were determined. Administration of *Ficus hirta* Vahl aqueous extract delayed the gastric emptying and small intestinal propulsion rates in a mouse model of spleen deficiency, thus enhancing digestion and absorption (Luo et al., 2012a). Consistent with the findings of previous studies, another study demonstrated that *Ficus hirta* Vahl has a two-way effect of strengthening and inhibiting small intestinal propulsion, which could improve gastrointestinal motility and promote digestion and absorption (Li et al., 2008).

In a further study, normal rats were used as control group, the researchers established a rat model of pyloric ligation-induced gastric mucosal damage and discovered that *Ficus hirta* Vahl aqueous extract (2 g/mL) reduced gastric acid secretion and pepsin activity in rats, and had protective effects against gastric mucosal injury (Wang et al., 2011a). In another study, indomethacin was subcutaneously injected into rats to establish a gastric mucosal injury model. Forty-two SD male rats were randomly divided into normal, model, and high-dose and low-dose groups (14 and 7 g/kg) *Ficus hirta* Vahl water extract groups. Gastric mucosal damage was observed in the model group, which was ameliorated in mice following intragastric administration of *Ficus hirta* Vahl The mechanism of action was found to be related to the effect of *Ficus hirta* Vahl on the expression of connexin and the transcription factor SOX2 (Zeng et al., 2020). In one study, 60 rats were randomly divided into six groups. The *Astragalus* water extract-treated rats acted as a control group and the *Ficus hirta* Vahl water extract groups were divided into high, medium, and low dose groups, with 10 rats in each group. Rats in the all groups except the control were subcutaneously injected with reserpine (1 mg/kg) daily to establish a spleen deficiency model. The spleen deficiency model group was intragastrically administered purified water at 10 mL/kg. The *Astragalus* water extract group and the high-, medium-, and low-dose *Ficus hirta* Vahl water extract groups were administered the corresponding drugs intragastrically at the same dose. The study showed that *Ficus hirta* Vahl aqueous extract increased the β-endorphin (β-EP), motilin (MTL) and gastrin (GAS) levels in the plasma of rats with spleen deficiency, thereby regulating the movement of the gastrointestinal tract (Yang and Xia, 2012). Therefore, the role of *Ficus hirta* Vahl in promoting the digestive system deserves attention.

### 6.3 Antitussive and antiasthmatic effects

Several studies have shown that *Ficus hirta* Vahl can be used to treat respiratory conditions, such as cough and asthma. The effects of the drugs on digestive tract motor function were measured using the intestinal propulsion method. The antitussive effect of the drug on mice was measured using a spray-induced cough model. The expectorant effect of the drug in mice was determined using tracheal segment secretion spectrophotometry. *Ficus hirta* Vahl improved gastrointestinal motor function, promoted digestion and absorption, had obvious cough-suppressant effects, had certain expectorant effects, and improved respiratory system function (Li et al., 2008). In one study, seventy-two mice, half male and half female, were randomly divided into six groups: control, positive control, *Astragalus* aqueous extract (10 g/kg), and *Ficus hirta* Vahl aqueous extract high (20 g/kg), medium (10 g/kg), and low (5 g/kg) dosage groups. The positive control group (10 mg/kg) was administered *Astragalus* aqueous extract intragastrically and the control group was administered an equal volume of distilled water for five consecutive days. *Ficus hirta* Vahl aqueous extract reduced the number of cough reactions induced by aqueous ammonia in mice, indicating that it had an antitussive effect. For the antiasthmatic experiment, guinea pigs were divided into six groups: normal control group, positive control group, *Astragalus* water extract group (8 g/kg), and *Ficus hirta* Vahl aqueous extract high (16 g/kg), medium (8 g/kg), and low (4 g/kg) dose groups. The treatments were administered continuously for 7 days. Different doses of *Ficus hirta* Vahl aqueous extract prolonged periods of asthma relief, indicating that *Ficus hirta* Vahl has an antiasthmatic effect (Zhou et al., 2009b). In this experiment, the vagus nerve in the afferent pathway of the cough reflex was stimulated with electricity to measure its threshold value for inducing cough. The results showed that the threshold value for inducing the cough voltage increased significantly with *Ficus hirta* Vahl treatment. The research showed that the ethanolic extract of *Ficus hirta* Vahl exhibited an antitussive effect. Research has demonstrated that *Ficus hirta* Vahl ethanol extract has potent anti-asthmatic effects and considerably extends the asthma incubation period (Zeng et al., 2002). According to recent clinical studies, *Ficus hirta* Vahl preparations can help reduce asthma symptoms (Dogra et al., 2015; N’guessan-Irié et al., 2020). This study demonstrated that *Ficus hirta* Vahl effectively reduced bronchiolitis in smoked + LPS chronic obstructive pulmonary disease model rats, as well as reducing cough, expectoration, and asthma in patients and preventing the decline of lung function (Zhou et al., 2008). Network pharmacology studies have revealed that *Ficus hirta* Vahl plays antitussive and antiasthmatic roles by acting on targets, such as estrogen receptor 1, epidermal growth factor receptor, cyclooxygenase, phosphatidylinositol, glycogen synthase kinase, and other signaling pathways (Huang and Huang, 2023). These investigations offer a foundation for the development of novel antitussive and antiasthmatic medications by elucidating the mechanism of action of *Ficus hirta* Vahl in relieving cough, decreasing phlegm, and relieving asthma.

### 6.4 Antibacterial effect

It is well known that *Ficus hirta* Vahl has antimicrobial properties. The minimum inhibitory concentration (MIC) of the *Ficus hirta* Vahl aqueous extract was 1 g/mL, which effectively inhibited the growth of pathogenic bacteria such as *E. coli*, *Bacillus subtilis*, and *S. aureus* (Wang et al., 2010a). Additionally, researchers discovered that *Ficus hirta* Vahl aqueous extract exhibited antibacterial activity against *Staphylococcus aureus* and *Escherichia coli*, with MICs of 11.21 and 14.07 mg/mL, respectively (Chen and Ye, 2012). These findings indicate that *Ficus hirta* Vahl possesses antibacterial activity, although its inhibitory effect varies among the different strains.

*Ficus hirta* Vahl has significant anti-*P. italiana* and anti-*Penicillium digitata* activity. Studies have shown that *Ficus hirta* Vahl causes cell death by destroying the membranes and cell walls of mycelia. The antifungal activity of the flavonone pinocembroside metabolite obtained from the *Ficus hirta* Vahl fruit was evaluated against *P. italicum*. Pinocembroside showed antifungal activity against the *in vitro* mycelial growth of *P. italicum*, with an MIC and minimum fungicidal concentration of 200 and 800 mg/L, respectively (Chen et al., 2020a; Chen et al., 2020b). A study showed that pinocembrin-7-O-β-D-glucoside was isolated from *Ficus hirta* Vahl, and it was discovered to be an effective concentration-dependent antibacterial metabolite (Xia et al., 2016; Chen et al., 2017). An *in vitro* experiment was conducted to evaluate the effects of *Ficus hirta* Vahl in LPS-induced apoptosis. In this study, Fas, an apoptosis receptor, was cloned, which included a 5′-UTR of 39 bp, an ORF of 951 bp, a protein of 316 amino acids, and a 3′-UTR of 845 bp. EcFas was the most strongly expressed in the spleen tissue of orange-spotted groupers. In addition, LPS-induced apoptosis in the fish spleen cells was concentration-dependent. These findings provide evidence that *Ficus hirta* Vahl treatment alleviates LPS-induced apoptosis by activating miR-411 to inhibit Fas expression and, therefore, provided possible strategies for bacterial infections in fish (Feng et al., 2024). These results indicate that *Ficus hirta* Vahl can destroy *Penicillium italiana* and *P. digitatum*. As a result, the above studies have verified the antibacterial activity of *Ficus hirta* Vahl, which is worthy of further development and utilization.

### 6.5 Anti-inflammatory effect

A mouse model of xylene-induced ear edema was used to study the anti-inflammatory effects of *Ficus hirta* Vahl Sixty male and female mice were randomly divided into the normal group, positive control group, and *Ficus hirta* Vahl aqueous extract high (20 g/kg), medium (10 g/kg), and low dose (5 g/kg) groups. Mice in the positive control group were administered 25 mg/kg indomethacin intragastrically, and the normal group was intragastrically administered the same volume of distilled water once daily for six consecutive days. Treatment with *Ficus hirta* Vahl aqueous extract considerably reduced mouse ear enlargement and the increased number of abdominal capillaries, thus showing anti-inflammatory effects (Zhou et al., 2008). The anti-inflammatory activity of bergapten in *Ficus hirta* Vahl was studied using color mass spectrometry, LCMS-IT-TOF, and GC-MS. In a zebrafish model of inflammation induced by cutting, bergapten significantly inhibited the recruitment of neutrophils and macrophages to the injured site, as well as the production and re-release of reactive oxygen species and NO. Bergamot may be helpful in the treatment of chronic inflammatory illnesses, according to a different study (Yang et al., 2018). *Ficus hirta* Vahl is frequently used to treat inflammatory diseases and nerve injuries because of its anti-inflammatory properties.

### 6.6 Anti-oxidant and anti-aging effects

Antioxidant effects are essential for human health because they can inhibit or reduce the damage caused by free radicals in cells. In one study, the polysaccharides, flavonoids, polyphenols, and amino acids in *Ficus hirta* Vahl demonstrated strong free radical scavenging ability, their IC_50_ values for 1, 1-diphenyl-2-picrylhdrazyl (DPPH) free radicals were 15.13, 103.72, 3.63, and 13.64 μg/mL, respectively (Kuang et al., 2015). The antioxidant capacities of luteolin, apigenin, and psoralen in *Ficus hirta* Vahl were determined using the DPPH method, and it was found that the antioxidant capacity of luteolin was the strongest (Yi et al., 2013). In a previous study, *in vitro* antioxidant activity test results showed that total coumarins from *Ficus hirta* Vahl have significant antioxidant activity. The median IC_50_ values of total coumarins from the root of *Ficus hirta* Vahl were 2.23 mg/mL and 0.33 mg/mL for DPPH free radicals and 2,2′-azino-bis(3-ethylbenzothiazoline-6-sulfonicacid) (ABTS) free radicals, respectively (Wang et al., 2023). According to one study, the antioxidant activity and total phenolic content of *Ficus hirta* Vahl in different leaf types varied, and the antioxidant activity and total phenolic content of *Ficus hirta* Vahl in the seven-finger type were significantly higher than those in the three- and five-finger types. Therefore, the total phenol content of plants is a significant indicator of the antioxidant capacity of *Ficus hirta* Vahl (Chen et al., 2021b; Qin et al., 2021).

Malondialdehyde (MDA) and superoxide dismutase (SOD) are recognized indicators of antioxidant drug-induced damage. A total of 100 mice were divided into five groups: normal control; model control; and *Ficus hirta* Vahl high-, medium-, and low-dose groups. The high-, medium-, and low-dose groups were administered *Ficus hirta* Vahl at doses of 2, 1, and 0.5 g/kg, respectively, for 9 consecutive days. Research found that *Ficus hirta* Vahl aqueous extract could prevent and treat fatigue in mice by improving SOD activity and the total antioxidant capacity of skeletal muscle, reducing MDA production, enhancing antioxidant capacity, and reducing oxidative stress injury (Luo et al., 2012b). To study the effects of different doses (36, 18, 9 g/kg/d) on memory function of Alzheimer’s disease (AD) mice, the study showed, using water maze experiments, that *Ficus hirta* Vahl could improve memory function and peroxide damage in AD mice by reducing MDA content and increasing SOD activity in brain tissue (Feng and Li, 2015).

Free radicals are recognized as primary culprits in the aging process. Owing to its rich antioxidant components, *Ficus hirta* Vahl extract can effectively scavenge free radicals. This antioxidant activity plays a crucial role in safeguarding cells from the detrimental effects of free radical damage, thereby preventing cellular senescence and ultimately delaying the overall aging process. Seventy experimental mice were randomly divided into 7 groups: normal group, model group, vitamin E (100 mg/g) group, *Ficus hirta* Vahl aqueous extract high (20 g/kg) and low (10 g/kg) dose groups, and *Ficus hirta* Vahl alcohol extract (20 g/kg) and low (10 g/kg) dose groups of. The index of the thymus and spleen of mice was calculated, and the MDA content and SOD activity in the serum, glutathione peroxidase (GSH-Px) activity in the liver, and catalase (CAT) activity in the brain were measured. Both the aqueous and alcohol extracts of *Ficus hirta* Vahl could increase thymus and spleen indices in the D-galactose-induced aging mouse model, increase SOD activity, GSH-Px activity, and CAT activity in aging mice to varying degrees, and reduce MDA content, which may be related to its antioxidant effect (Wang et al., 2018; Ye et al., 2017).

### 6.7 Hepatoprotective effect

*Ficus hirta* Vahl has hepatoprotective effects against acute and chronic liver injury, alcoholic liver injury, non-alcoholic fatty liver disease, liver fibrosis, and liver cancer (Yang et al., 2024). *Ficus hirta* Vahl protected the liver of mice with acute liver injury caused by cocaine, xylene, and dimethylformamide. Following the injection of *Ficus hirta* Vahl aqueous extract, mouse liver tissue showed reduced pathological damage and lower levels of serum transaminases (alanine transaminase [ALT] and aspartate transaminase [AST]), CAT, and other enzymes, suggesting that *Ficus hirta* Vahl can reduce these enzymes and withstand lipid peroxidation (Cai et al., 2007; Zhou et al., 2008). In another study, *Ficus hirta* Vahl aqueous extract exhibited a protective effect against restraint stress-induced liver injury in mice. The aqueous extract of *Ficus hirta* Vahl has the potential to significantly reduce plasma ALT activity, liver tissue MDA, and NO content; significantly increase liver tissue oxidative free radical absorption capacity and glutathione (GSH) content; and improve glutathione peroxidase (GSH-Px) and glutathione S-transferase activity. This experiment verified that *Ficus hirta* Vahl protected the liver by reducing oxidative stress (Wang et al., 2015). Carbon tetrachloride (CCL_4_)-induced liver injury is a commonly used model of acute liver injury. For the first time, LC-MS-based metabolomics was used to elucidate the hepatoprotective effects of *Ficus hirta* Vahl on CCl_4_-induced acute liver injury in mice. The mechanism participates in the regulation of oxidative stress and energy homeostasis, and reverses the metabolomic characteristics. In particular, the metabolism of amino and fatty acids can be improved by *Ficus hirta* Vahl pretreatment (Zhou et al., 2022). This provides insights into potential metabolic mechanisms for future research.

Oxidative stress is considered the main cause of alcoholic liver disease (Li et al., 2015). As alcohol metabolism produces a large amount of ROS and active nitrogen free radicals, it causes oxidative stress damage to the liver when it exceeds the body’s ability to remove them (Neuman et al., 2015). Researchers have studied the effect of the alcohol extract of *Ficus hirta* Vahl on alcoholic liver injury in mice, and the results have shown that *Ficus hirta* Vahl alcohol extract significantly increases the activity of antioxidant enzymes in the liver. The mechanism may be to activate Nrf2, induce the regulation of downstream antioxidant factors or antioxidant systems, inhibit the abnormal activation of CYP2E1, reduce the oxidative stress response, and thereby reduce alcohol damage in the livers of mice (Zhang et al., 2021). Besides, a study revealed that the protective mechanism of *Ficus hirta* Vahl against alcoholic liver injury involves decreasing the levels of inflammatory factors such as NF-κB, tumor necrosis factor (TNF)-α, IL-1β, and IL-6 while increasing the levels of GSH and SOD (Zhang and Du, 2021b). In another study, *Ficus hirta* Vahl extract reduced alcohol-induced liver damage by lowering the levels of inflammatory markers such as IL-6, IL-1β, TNF-α, and NF-κB in liver tissue, which may be connected to coumarin and flavonoid contents (Zhang and Du, 2021a). In addition, the study found that aqueous and alcohol extracts of *Ficus hirta* Vahl could reduce the activity of ALT and AST to different degrees, indicating that *Ficus hirta* Vahl can reduce liver damage caused by alcohol and protect the liver. Further studies have found that *Ficus hirta* Vahl could reduce the levels of MDA, the product of lipid peroxidation, and increase SOD and GSH levels, GSH-Px activity, and the expression levels of Nrf2 and its downstream related antioxidant enzymes HO-1 and SOD-1 proteins to play an antioxidant role (Du, 2021). In another study, *Ficus hirta* Vahl to was administered to alcohol-induced liver injury model mice. As a result, the pathological changes in liver lipids and inflammatory cell infiltration were reduced, the concentrations of AST, ALT, alkaline phosphatase, and lactate dehydrogenase were significantly reduced, and the liver sinuses were normal. It was concluded that *Ficus hirta* Vahl extract had obvious preventive and treatment effects on alcohol-induced liver injury in mice (Feng et al., 2018). *Ficus hirta* Vahl also has preventive and therapeutic effects on non-alcoholic liver disease, mainly through the regulation of lipid metabolism and inflammation. In the process of lipid metabolism regulation, CD36, SREBP-1, SCD1, PPARγ, ACOX1, and CPT1α are the key factors in *Ficus hirta* Vahl treatment of nonalcoholic fatty liver disease. It was found that SREBP-1, ACACA, and SCD1 levels were significantly reduced, while those of CPT1α, ACOX1, and PPARα were significantly increased. During the inflammatory process, the downregulation of IL-6, IL-1β, and TNF-α is involved in the remission of nonalcoholic fatty liver disease (Quan et al., 2022).

**FIGURE 11:**
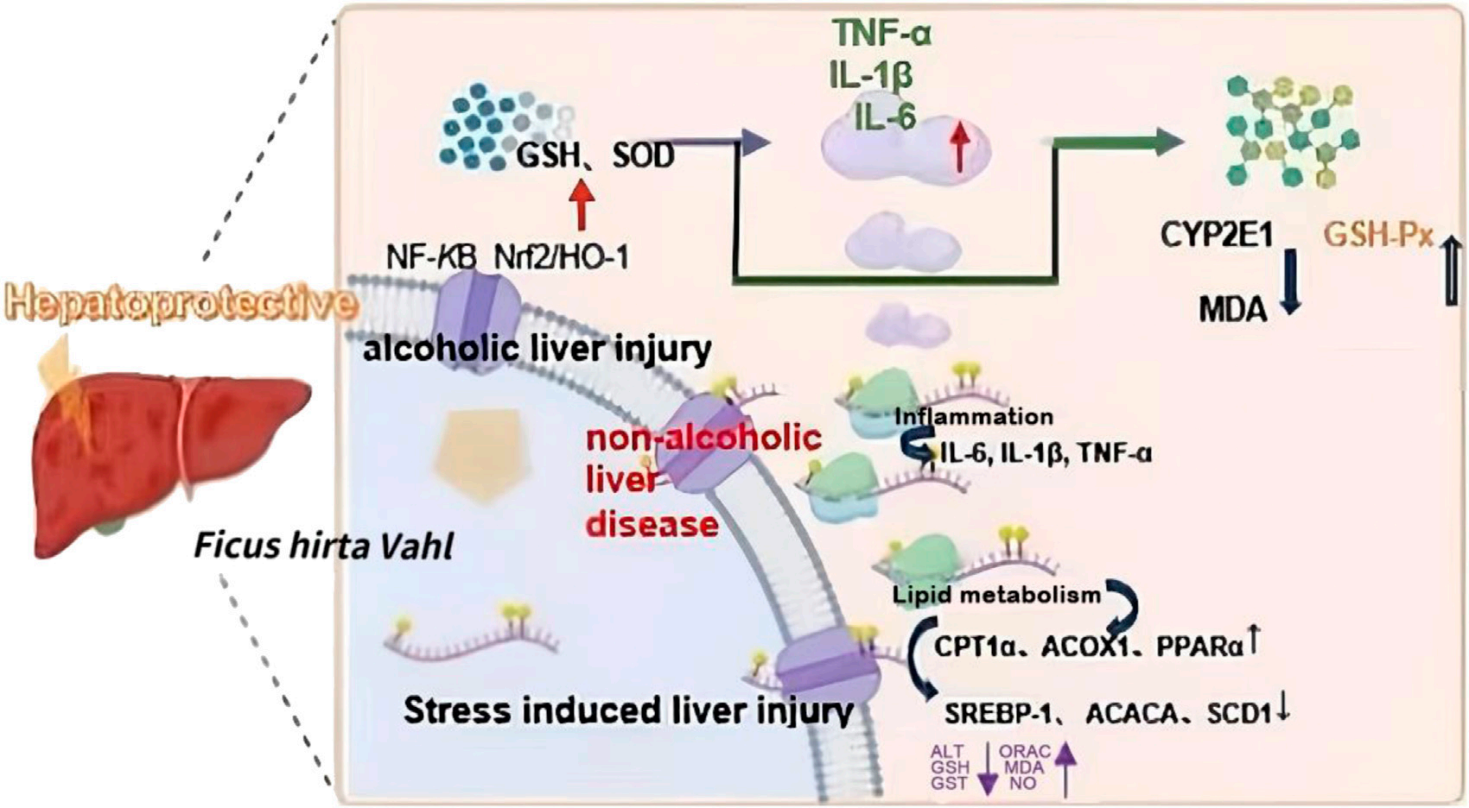
Part of the hepatoprotective mechanism of *Ficus hirta* Vahl is shown in this figure. Abbreviations: nuclear factor kappa-B (NF-κB); Nuclear factor erythropoietin-2-related factor 2/heme oxygenase 1 (Nrf2/HO-1); glutathione (GSH); Superoxide dismutase (SOD); tumor necrosis factor α (TNF-α); interleukin-1β (IL-1β); interleukin-6 (IL-6); Cytochrome P450 Family 2 Subfamily E Member 1 (CYP2E1); Malondialdehyde (MDA); Glutathione peroxidase (GSH-Px); Carnitine palmitoyltransferase 1α (CPT1α); Acyl-CoA Oxidase 1 (ACOX1); Peroxisome proliferator-activated receptor-α (PPARα); Sterol regulatory element-binding proteins (SREBP-1); Acetyl-CoA Carboxylase Alpha (ACACA); Stearoyl-CoA desaturase-1 (SCD1); alanine aminotransferase (ALT); nitric oxide (NO); oxygen radical absorbance capacity (ORAC); Glutathione-s-transferase (GST) (created by Figdraw: https://www.figdraw.com).

Furthermore, many TCM prescriptions for the clinical treatment of liver illnesses include *Ficus hirta* Vahl, suggesting that its importance in liver protection cannot be overlooked according to the currently available literature (Qiu et al., 2012). Moreover, *Ficus hirta* Vahl decoction has been found to improve clinical signs, liver function, and liver fibrosis in patients with post-hepatitis B cirrhosis (You et al., 2011). Tea bags made with *Ficus hirta* Vahl are suitable for people with hepatitis B. *Ficus hirta* Vahl can improve immunity, promote antibody production, strengthen liver protection, and prevent the reduction of liver glycogen levels (Li, 2005). Thus, these studies suggest that *Ficus hirta* Vahl has the potential to develop into a hepatinica. Part of the hepatoprotective mechanism of *Ficus hirta* Vahl is shown in Figure 11.

### 6.8 Radiation-protective effect

The radiation-protective potential of *Ficus hirta* Vahl extracts was investigated. Psoralen in *Ficus hirta* Vahl can improve the survival rate of mice with bone marrow injury and plays an effective anti-radiation role in radiation damage to hematopoietic tissue (Zhang et al., 2024). Several studies revealed that *Ficus hirta* Vahl had protective and repairable anti-radiation effects on the DNA of bone marrow and lung tissues in mice damaged by 60Coγ rays (Wang et al., 2010b; Wang et al., 2011b; Wang et al., 2010c).

### 6.9 Antitumour effect

**FIGURE 12:**
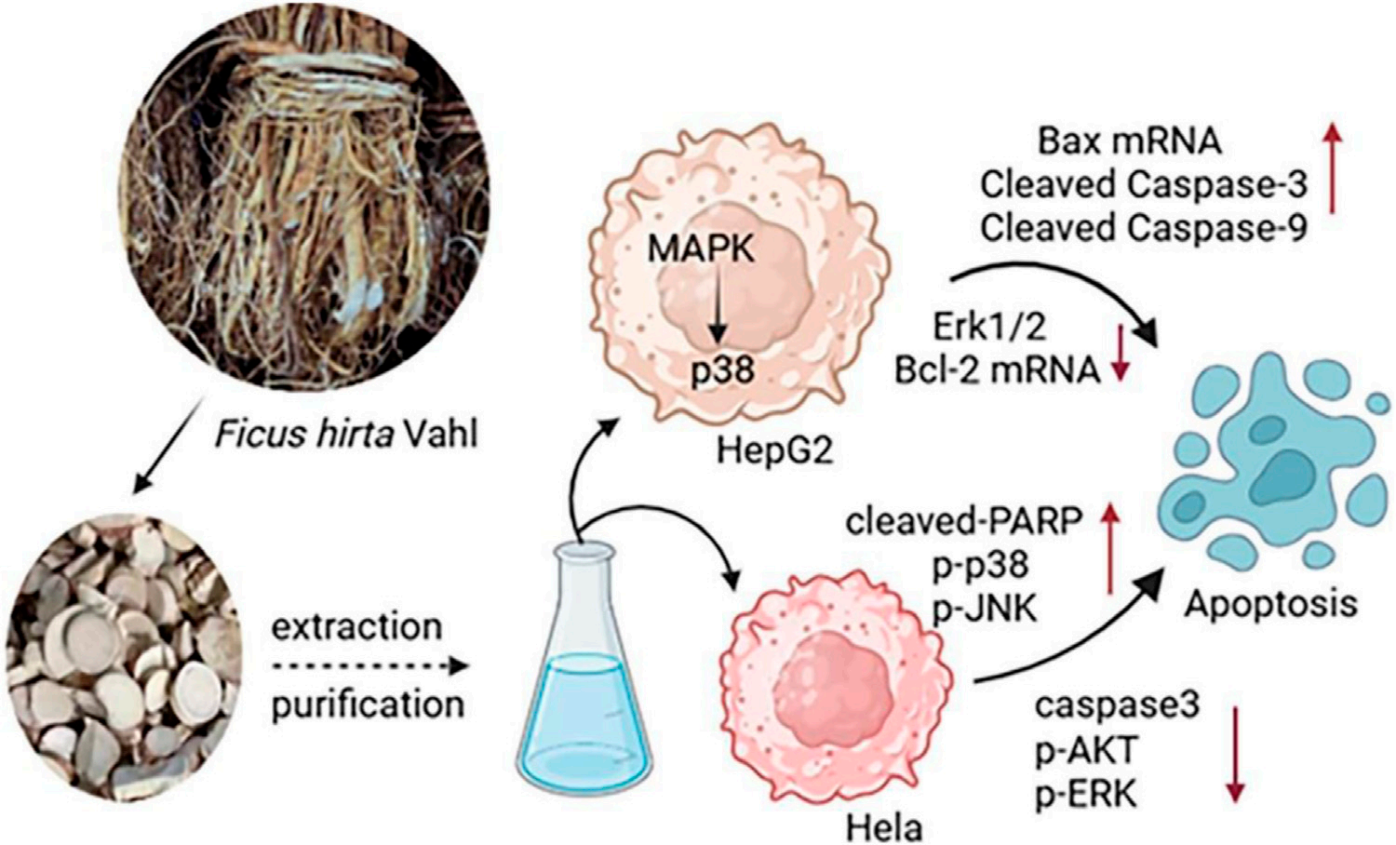
Part of the antitumous mechanism of *Ficus hirta* Vahl is shown in this figure. Abbreviations: mitogen-activated protein kinase (MAPK); human hepatocellular carcinomas (HepG2); extracellular signal-regulated kinase 1/2 (Erk1/2); B-cell lymphoma-2 (Bcl-2); cleaved-Poly ADP-ribose polymerase (cleaved-PARP); Phospho-p38 (p-p38); Phospho-c-Jun N-terminal kinase (p-JNK); cysteinyl aspartate specific proteinase3 (caspase3); Phospho-protein kinase B (p-AKT); Phospho-ERK (p-ERK) (created by BioRender: https://biorender.com/).

*Ficus hirta* Vahl has shown promising results as an antitumor botanical drug. An *Ficus hirta* Vahl extract inhibited the proliferation of HepG2 cells, decreased the mRNA and protein expression of Bcl-2, and increased the mRNA and protein expression of Bax, thereby accelerating the apoptosis of cancer cells (Yang et al., 2019; Cen et al., 2010). In one study, extracts of *Ficus hirta* Vahl had a strong cytotoxic effect on HepG2 cells, inducing apoptosis in HepG2 cells through the JNK and p38MAPK signaling pathways. In another study, the MTT assay was used to detect the cytotoxic activity of *Ficus hirta* Vahl against HeLa, MCF-7, and H460 cell lines, in which the isoprene flavonoids inhibited the proliferation of HeLa cells with an IC_50_ value of 28.88 μM. HeLa cell apoptosis is induced through the MAPK and AKT signaling pathways (Ye et al., 2022). Water, ethyl acetate, and n-butanol *Ficus hirta* Vahl extracts inhibited HeLa cell proliferation and induced HeLa cell apoptosis. The extracts decreased the viability of HeLa cells in a dose-dependent manner and induced a sub-G1 peak in the flow cytometry histogram of treated cells compared to the control (Zeng et al., 2012). A previous study revealed that psoralen inhibited the proliferation of gastric cancer BGC-803 cells and induced tumor cell apoptosis (Yan et al., 2015). It also inhibits the proliferation of the human breast cancer MCF-7 cell line (Jiang et al., 2016). Part of the antitumous mechanism of *Ficus hirta* Vahl is shown in Figure 12.

Additionally, *Endomelanconiopsis*, an endophytic fungal strain isolated from *Ficus hirta* Vahl, has anti-tumor activity (Liang, 2014). However, in another study on *Ficus hirta* Vahl endophytic fungi, no metabolites exhibited anticytotoxic activity against SF-268, MCF-7, NCI-H460, or HepG2 cells (Sun et al., 2016). Therefore, it remains to be determined whether *Ficus hirta* Vahl endophytic fungi exert anti-tumor effects.

## 7 Nutritional composition and other potential uses of *Ficus hirta* Vahl

### 7.1 Nutritional value and the food industry

*Ficus hirta* Vahl is a TCM with value as both medicine and food. It has high nutritional value and is rich in minerals, vitamins, amino acids, and plant proteins. It is also a plant that has been investigated by nutritionists. *Ficus hirta* Vahl, often known as “Canton ginseng,” is a popular soup ingredient in the Canton and Guangxi areas of China due to its therapeutic effects. *Ficus hirta* Vahl soup smells strongly like botanical drugs and also smells somewhat similar to coconut. It can be used as a seasoning for cooking a variety of dishes. People also use *Ficus hirta* Vahl to make wine, which is golden in color, fragrant, sweet, mellow in flavor, and has a well-balanced medicinal taste. *Ficus hirta* Vahl not only has good health-promoting and curative effects, but also has high food and drug value. Some studies have reported the brewing process steps of *Ficus hirta* Vahl sweet wine and medicinal liquor (Kuang, 1999), which provide a basis for the development and utilization of *Ficus hirta* Vahl medicinal liquor.

A list of products, including candies, drinks, and tea substitutes, that use *Ficus hirta* Vahl as a raw ingredient has been published (Zhang et al., 2009; Yue, 2021). Studies have indicated that essential nutrients, such as amino acids, organic acids, carbohydrates, and volatile fragrance metabolites, are present in *Ficus hirta* Vahl Amino acid content and composition can be used as indicators to evaluate the nutritional and health value of *Ficus hirta* Vahl (Liu et al., 2014). Both essential and nonessential amino acids are present in *Ficus hirta* Vahl According to previous research, the non-essential amino acids in *Ficus hirta* Vahl are L-arginine (4.549 mg/g), L-alanine (3.989 mg/g), and L-proline (3.818 mg/g) (Du et al., 2023). *Ficus hirta* Vahl is rich in minerals, such as Ca, K, Mg, Fe, Cu, Zn, Mg, Mn, and Pb. It also contains large amounts of vitamins, of which vitamin C is the most abundant (Su et al., 2007). A study showed that *Ficus hirta* Vahl polysaccharide is a key metabolite that regulates the function of intestinal flora, revealing the potential of *Ficus hirta* Vahl polysaccharide as a natural and effective prebiotic (Xiao, 2022). Therefore, *Ficus hirta* Vahl polysaccharide can be used as a natural new functional food metabolite or biological material, and the application of *Ficus hirta* Vahl in the development of functional beverages, dietary supplements, and pharmaceutical preparations is deepened.

Moreover, the flesh of *Ficus hirta* Vahl fruit is a low-fat, low-calorie food that can be consumed by individuals who want to maintain a good blood lipid profile and weight. Other studies have shown that the four different split-leaf *Ficus hirta* Vahl fruits, including whole leaves (pinnate leaf and intact leaf), three split leaves (palmate leaves deeply split into three pieces), five split leaves (palmate leaf deeply split into five pieces) and 7-lobed leaf (palmate leaf deeply split into seven pieces), are rich in amino acids. The fruit of *Ficus hirta* Vahl may be developed as a medicinal plant with a high protein content, making it more suitable for further processing and utilization (Chen et al., 2024).

Because of its potential nutritional and therapeutic properties, *Ficus hirta* Vahl has become increasingly popular in modern society. Researchers have used *Ficus hirta* Vahl juice (30%), sugar (16%), jelly powder, and water as raw materials to develop a fragrant and resilient nutritional health jelly, which has opened up a new way for the development of functional health foods (Huang et al., 2009). In a study on the relationship between the composition and metabolic function of the human intestinal microflora and the *Ficus hirta* Vahl diet, it was found that the *Ficus hirta* Vahl diet changed the fermentation of carbohydrates by intestinal microorganisms, resulting in more short-chain fatty acids, which were more abundant in amino acid synthesis and the citric acid cycle (Xiao et al., 2022). These results indicate that *Ficus hirta* Vahl diet can optimize the structure and metabolism of the intestinal microbial community and form a healthy internal environment.

*Ficus hirta* Vahl and its pharmaceutical preparations, such as *Ficus hirta* Vahl granules, Xichuan pills, *Ficus hirta* Vahl Yangxue Fuzheng Decoction, *Ficus hirta* Vahl Huoxue Yangyan Decoction, can be used in the treatment of digestive, liver, and respiratory diseases in clinical practice,. The production of medicinal powders and decoctions is worthy of market promotion, which not only maintains the nature and taste of the TCM decoction, but also melts quickly, makes full use of its medicinal activity, and further optimizes its application value. It is also promising to formulate *Ficus hirta* Vahl into a tablet with healthcare functions.

### 7.2 Agriculture

The bacteriostatic effects of *Ficus hirta* Vahl have also been exploited for the production of plant pesticides (Xing et al., 2022). Citrus rot is commonly caused by *P. italiana* and *Penicillium digitum*. *Ficus hirta* Vahl has a clear inhibitory effect on *Penicillium* growth and infection (Chen, 2021). A study found that (2R) methyl 2-o-β-D-glucopyrano2-phenylacetate was isolated from *Ficus hirta* Vahl fruit, which showed antibacterial activity against *P. italiana*, indicating that it may be a potential natural fungicide (Wan et al., 2016; Le et al., 2021; Ye et al., 2020). Therefore, it is anticipated that *Ficus hirta* Vahl can be used to develop natural preservatives for citrus. Another study showed that *Ficus hirta* Vahl aqueous extract was added to chitosan and coated on the surface of citrus. This reduced the decay, weight loss, and respiration rates, and the MDA content of the fruit. Thus, the content of ascorbic acid and the activity of peroxidase in fruit were reduced, reducing oxidative stress and delaying the effect of lipid peroxidation to improve fruit preservation (Chen et al., 2019).

The success or failure of shrimp farming largely depends on the quality of the water used for aquaculture. Ammonia nitrogen and pH are important indicators of aquaculture water quality. Ammonia nitrogen is produced by the decomposition of nitrogen-containing organic matter such as shrimp residue and excrement (Drzeżdżon et al., 2018). The breakdown of organic waste containing nitrogen, such as shrimp leftovers and excrement, produces ammonia nitrogen. Shrimp immunity, susceptibility to pathogens, and the ability to withstand disease are all compromised by high levels of ammonia and nitrogen stress, endangering their survival and ability to thrive. Nitrogen and oxygen stresses are common environmental stress factors in shrimp cultures. To address this problem, it has been found that supplementing shrimp feed with *Ficus hirta* Vahl can improve the antioxidant capacity and ammonia nitrogen tolerance of shrimp. These effects are mediated by reducing excessive ROS by inhibiting the expression of miR-2765 in shrimp and activating antioxidant enzyme activity (Liang et al., 2021). In conclusion, *Ficus hirta* Vahl offers a novel approach to enhance the aquatic habitat of shrimp raised for aquaculture and merits further investigation and advancement.

## 8 Breeding and cultivation technology

Market demand for *Ficus hirta* Vahl increases as more individuals become aware of the medical value and health benefits of the plant. Therefore, *Ficus hirta* Vahl cultivation technology is urgently required for sustainable planting. Cultivation technology is the 'cornerstone’ of the *Ficus hirta* Vahl planting industry. Selecting seedlings, planting, managing weeds, pruning, controlling pests, harvesting, and other related tasks are essential (Li et al., 2023d). There are numerous advantages to developing and planting *Ficus hirta* Vahl beneath the forest, however, due to *Ficus hirta* Vahl seeds being small, the seeds can be combined with plant ash prior to planting. The ideal temperature for raising seedling is 25°C–30°C. (Dong et al., 2014). The effects of different ratios of N, P, and K fertilizers on the yield of *Ficus hirta* Vahl were studied. It was found that the optimum fertilization was 145.8 kg/h m^2^ for N, 83.2 kg/h m^2^ for phosphorus (P_2_O_5_), and 186.9 kg/h m^2^ for potassium (K_2_O). The yield-increasing effects were N, P, and K fertilizers, with yield-increasing rates of 78.2%, 69.5%, and 54.6%, respectively (Li and Wang, 2020). Innovations in cultivation techniques and new agricultural technologies, such as hydrogen water treatment, can increase the phenylpropanoid and flavonoid contents. In the future, efficacy may be enhanced directionally by regulating plant hormone signals. In summary, *Ficus hirta* Vahl has the potential for sustainable agricultural development.

## 9 Toxicology

To assess the safety of *Ficus hirta* Vahl, researchers observed the death, activity, nutrition, body weight, anatomical organs, and pathological examination of mice using the acute toxicity test method. The maximum dosage of this experiment was 370 times the human dosage, which showed the safety of the clinical daily dosage of the raw drug at 50 g/d. The results showed that there was no death in the mice, and there was no significant difference in activity, diet, body weight, anatomical organs, or pathological examination compared to the control group (Luo et al., 2009). It is well known that pesticide residues are an important factor affecting drug safety. Pesticide residues in *Ficus hirta* Vahl were determined using GC-MS. The pesticide residues in *Ficus hirta* Vahl samples were minimal, with the exception of γ-666, which had a residue of 0.0005 mg/kg, and the rest were not detected (Zhen et al., 2018). These findings provide a theoretical basis for safe use of *Ficus hirta* Vahl, as it is a demonstrably safe medicinal plant. Of course, it is necessary to pay attention to the choice of dosage and the compatibility relationship to make more rational use of this resource.

## 10 Identification methods

Plant misidentification is a common problem during the identification of TCM. Therefore, an energy-saving onsite identification method was developed to quickly identify *Ficus hirta* Vahl Researchers can use a rapid DNA extraction scheme called Recombinase Polymerase Amplification of DNA Barcoding (Tian et al., 2017). *Ficus hirta* Vahl may not only be confused with different species, but also with plants of the same genus. Researchers have created DNA barcodes for *Ficus hirta* Vahl (Vu et al., 2023). This provides a basis for the accurate and comprehensive identification of *Ficus hirta* Vahl.

## 11 Discussion

*Ficus hirta* Vahl, a plant with medicinal and edible properties, has a significant value in traditional medicine and modern research. Based on the main findings and their implications, *Ficus hirta* Vahl has diverse characteristics and potential applications. In terms of botanical characteristics, their unique morphologies and wide distributions provide a basis for resource development. In traditional medicine, *Ficus hirta* Vahl is used to treat a variety of diseases, covering multiple fields such as the digestive system, respiratory system, and liver diseases. This reflects its important position in folk healthcare and provides a theoretical basis for modern drug research and development.

Modern studies have isolated 130 metabolites from *Ficus hirta* Vahl, such as flavonoids and phenylpropanoids. These metabolites endow *Ficus hirta* Vahl with multiple biological activities and play roles in immunoregulation, digestion promotion, and antibacterial and anti-inflammatory effects, providing a scientific basis for its applications in the medical, food, and agricultural fields. In terms of nutritional components, *Ficus hirta* Vahl is rich in minerals and vitamins and can be developed as a nutritional supplement. At the same time, its role in improving the intestinal flora provides a direction for the research and development of functional foods. In agriculture, the antibacterial properties of *Ficus hirta* Vahl can be used for plant pesticides and fruit preservation. It can also improve the shrimp farming environment, which has positive implications for the sustainable development of agriculture.

However, there are still some gaps in current research on *Ficus hirta* Vahl Although numerous metabolites have been identified, relatively few studies have been conducted on their chemical properties and pharmacological effects. There is a lack of in-depth research on specific metabolites, and unique plant metabolites have not yet been reported. In terms of biological activity, most studies have focused on extracts, and the pharmacological mechanisms of specific metabolites remain unclear. In addition, research on the mechanism of action and pharmacokinetics of *Ficus hirta* Vahl is insufficient, and the lack of relevant information limits its further development and utilization. Although it has a wide range of traditional applications, data from large-scale clinical trials are lacking.

In the future, it will be necessary to strengthen in-depth research on *Ficus hirta* Vahl metabolites and clarify their pharmacological mechanisms and pharmacokinetic characteristics. Bottlenecks in clinical translation exist, although there are abundant achievements in in vitro and animal experiments, there is a lack of large - scale clinical trial data. Further high-quality clinical studies should be conducted to verify its efficacy and safety. At the same time, formulation technology should be optimized to improve the bioavailability of active ingredients and promote the sustainable development and application of *Ficus hirta* Vahl in the medical, food, agricultural, and other fields.

## 12 Conclusion and prospects

*Ficus hirta* Vahl is a TCM notable value for both its medicinal qualities and use as a food. Ancient records, experimental research, and clinical practice have shown that *Ficus hirta* Vahl is widely used for the treatment of dyspepsia, stomach disease, hepatitis, rheumatism, edema, *postpartum* agalactia, respiratory diseases, gynecological disorders, and pediatric diseases. Research has shown that it has a significant therapeutic value and is safe. Despite the abundance of ethnopharmacological documentation on *Ficus hirta* Vahl, there is insufficient scientific research. Ancient literature indicates that *Ficus hirta* Vahl can be used to treat hematuria, anal prolapse, eczema, and urticaria. This suggests that it has potential applications for the treatment of urinary and skin diseases. However, related experimental studies are scarce.

A total of 130 metabolites were isolated from *Ficus hirta* Vahl The flavonoids possess various biological activities, including antioxidant, anti-inflammatory, antibacterial, antiviral, and antitumor effects. The most effective flavonoids in *Ficus hirta* Vahl possess unique pharmacological properties. Among the flavonoid metabolites, apigenin and hesperidin are responsible for their antioxidant and immunomodulatory effects. Among the phenylpropanoid metabolites, psoralen and bergapten play a leading role in anti - inflammatory and antitumor effects. Although many phytochemicals have been isolated from *Ficus hirta* Vahl, there have been few studies on their chemical properties and pharmacological effects. Unique plant metabolites in *Ficus hirta* Vahl have not yet been reported, and whether there are specific unique plant metabolites in *Ficus hirta* Vahl requires further study. In terms of bioactivity, extracts from plant species have been shown to regulate the immune system, promote digestive system function, and exhibit antitussive, antiasthmatic, hepatoprotective, and radiation-protective properties. However, most of these compounds are concentrated in the extracts and the pharmacological effects of their specific metabolites are not clear. Therefore, it is necessary to elucidate the pharmacological effects of these metabolites. In addition, research on its mechanism of action is insufficient and lacks information on its pharmacokinetics; therefore, it is necessary to study its mechanism of action and pharmacokinetics in depth.

## Abbreviations

TCM, traditional Chinese medicine; ODS, Octadecylsilyl; HPLC, High Performance Liquid Chromatography; UV, ultraviolet spectroscopy; IR, infrared spectroscopy; NMR, Nuclear Magnetic Resonance; GC, gas chromatography; MS, mass spectrometry; ESI, electrospray ionization; 1D-NMR, one-dimensional nuclear magnetic resonance; 2D-NMR, two-dimensional nuclear magnetic resonance; HRESI-MS, high-resolution electrospray ionization mass spectrometry; UPLC, Ultra Performance Liquid Chromatography; LPS, Lipopolysaccharides; MSD, Mean Square Displacement; DRP1, dynamin-related protein 1; SCFA, CTX MAPK1 STAT3 MAPK3 AKT1 STAT1 NSAID SOX2 Short-chain fatty acids Cyclophosphamide mitogen-activated protein kinase 1 signal transducer and activator of transcription 3 mitogen-activated protein kinase 3 protein kinase B 1 signal transducer and activator of transcription Nonsteroidal Antiinflammatory Drugs SRY-Box Transcription Factor 2; IC_50_, Half of Inhibition Concentration; 5-LOX, 5-Lipoxygenase; LCMS-IT-TOF, Liquid chromatography-mass spectrometry–ion trap–time of flight; MDA, Malondialdehyde; SOD, Superoxide Dismutase; GSH, Glutathione; GSH-Px, Glutathione peroxidase; DNA, Deoxyribonucleic acid.
